# Enhancing Superexchange through Frontier Orbital Engineering in a van der Waals Metal–Organic Magnet

**DOI:** 10.1021/acs.chemmater.6c00516

**Published:** 2026-05-26

**Authors:** Jem Pitcairn, Mario Antonio T. Ongkiko, Peter J. Speakman, Jeremiah P. Tidey, Jack W. Jordan, Graham N. Newton, Pascal Manuel, J. Ross Stewart, Andrew J. Morris, Matthew J. Cliffe

**Affiliations:** † School of Chemistry, 6123 University of Nottingham, University Park, Nottingham NG7 2RD, United Kingdom; ‡ School of Metallurgy and Materials, University of Birmingham, Birmingham B15 2TT, United Kingdom; § Department of Physics, 2707 University of Warwick, Gibbet Hill Road, Coventry CV4 7AL, United Kingdom; ∥ GSK Carbon Neutral Laboratories for Sustainable Chemistry, School of Chemistry, University of Nottingham, Jubilee Campus, Nottingham NG7 2GT, United Kingdom; ⊥ 120797 ISIS Neutron and Muon Source, STFC Rutherford Appleton Laboratory, Didcot OX11 0QX, United Kingdom; # Department of Materials Science and Metallurgy, 2152 University of Cambridge, 27 Charles Babbage Road, Cambridge CB3 OFS, United Kingdom

## Abstract

van der Waals metal–organic magnets (vdW MOMs) offer the possibility of realizing a wide range of magnetic functionalities not possible in inorganic vdW magnets. In particular, their modularity allows for specific control over ligand-mediated superexchange and hence their ground states. We report a new vdW MOM, CrCl_2_(btd) (btd = 2,1,3-benzothiadiazole), assembled from a high-energy early transition metal and a redox-active ligand, which leads to a small charge-transfer gap. We solve the structure using a combination of powder neutron diffraction and single-crystal electron diffraction, showing that this material contains Cr^2+^btd^0^. Magnetometry, neutron diffraction, and inelastic neutron scattering measurements allow us to quantitatively determine the magnetic properties of this vdW magnet, showing it is a collinear rectangular antiferromagnet with small easy-axis anisotropy (*J*_Cl_ = −15.31(5) K, *J*_btd_ = −6.96(10) K, *D* = −2.0(2) K). Critically, we find that the superexchange through btd, with a low-lying LUMO, is significantly stronger than in the CrCl_2_(pym) (pym = pyrimidine) analogue (*J*_pym_ = +1.2(2) K), demonstrating that exchange can be enhanced by potentially redox-active ligands without actual electron-transfer onto ligands. This work provides a route toward vdW antiferromagnetic MOMs with strong magnetic superexchange.

## Graphical abstract

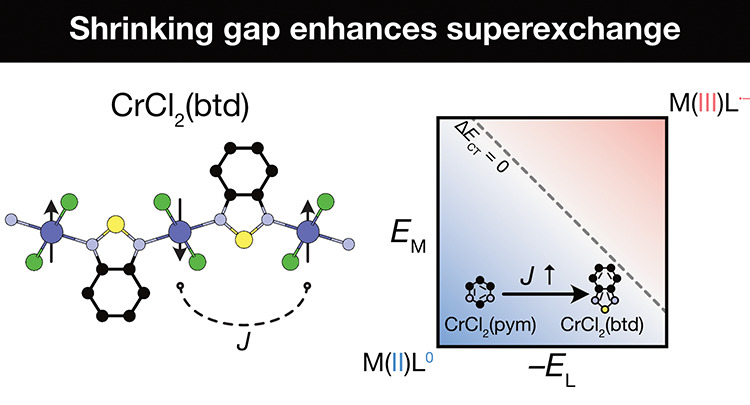

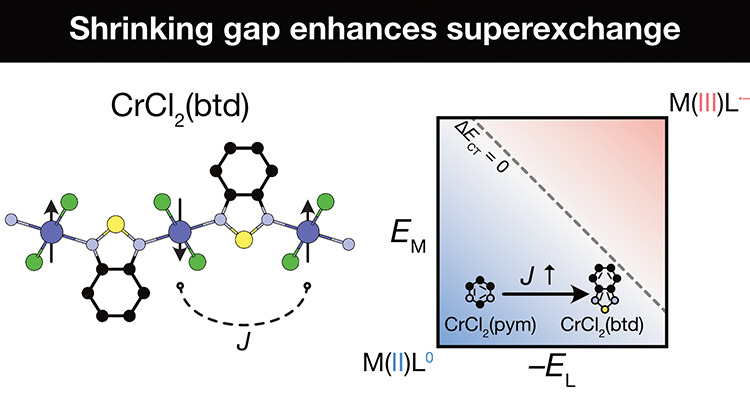

## Introduction

Metal–organic magnets (MOMs) are extended networks of metal nodes connected by organic molecular linkers.^1^ MOMs offer additional options for chemical design compared to traditional inorganic magnets: there is a wide variety of organic ligands, and modularity allows tuning of both structure and property while preserving network topology.^2–6^ Organic ligands are also particularly effective for realizing lower-dimensional structures, and hence the chemical diversity afforded in MOMs can also be used to dramatically expand the range of functional properties available in vdW magnets.^2,4^

Magnetic superexchange mediated by organic linkers is, however, typically weak. This weak magnetic coupling enables low-dimensional quantum magnetism in materials with a higher structural dimensionality.^7,8^ However, this weak through-ligand exchange also makes realizing cooperative magnetism at room temperature challenging, restricting their practical utility, e.g., in lightweight magnets^9^ or spintronics.^10,11^

Several promising strategies have emerged for designing strongly interacting MOMs.^1,12^ The two key factors are increasing metal–ligand orbital spatial overlap and minimizing the gap between metal and ligand orbital energies, Δ_CT_.^13–16^ Indeed, where Δ_CT_ becomes negative, and the overlap is larger, charge, and hence spin, can be transferred onto the ligand. These radical ligands, vdW MOMs, can have drastically enhanced interaction strength and even produce ferromagnetism or ferrimagnetism at room temperature.^8,9^ While these materials are of particular current excitement, the radical ligand approach cannot produce antiferromagnets, and hence important states such as frustrated spin liquid states;^17^ altermagnetism;^18^ and fast switching spintronic devices.^19^ MOMs with positive but small Δ_CT_ will not form radical ligands but can still yield strong superexchange, and early transition metals, with high energy and diffuse d-orbitals, are therefore a promising route for realizing this.^13,20^

In our previous work, we reported CrCl_2_(pyrimidine), a van der Waals MOM formed from CrCl_2_ chains bridged by ligands into two-dimensional (2D) sheets, which has comparatively weak superexchange through the organic ligand (*J*_pym_ = +1.2(2) K), despite the use of an early transition metal and a π-delocalized N-heterocyclic ligand.^7^ Previous work has also established that MCl_2_(btd), 2,1,3-benzothiadiazole (btd), M = Fe, Co, Ni, and Cu, adopts analogous structures to MCl_2_(pym) with similar magnetic properties between the two ligands.^2,21,22^ However, a key difference between the pym and btd is that btd can be readily reduced, with a low-energy lowest unoccupied molecular orbital (LUMO).^23^ This low-energy LUMO, when combined with the reducing early transition metals, could thus significantly enhance magnetic superexchange, even without reduction of the ligand.

Here we report CrCl_2_(btd), a 2D layered metal–organic magnet consisting of CrCl_2_ chains bridged by btd ligands to form a structure analogous to that of the other transition metal btd chlorides.^2,21,22^ We first describe its synthesis and structural characterization using powder diffraction and single-crystal electron diffraction, confirming the Cr^2+^(btd)^0^ oxidation states through the Jahn–Teller (JT) distortion. We then show through bulk magnetization, powder neutron diffraction (PND), and powder inelastic neutron scattering (INS) measurements that CrCl_2_(btd) orders into a Néel ground state at *T*_N_ = 46(2) K, with antiferromagnetic (AFM) ordering along the CrCl_2_ chain, AFM coupling of the chains through btd, and interlayer AFM correlations. Through a thorough analysis of the INS data, we quantitatively determine the energies of the key magnetic interactions and, in conjunction with bulk susceptibility measurements, show that CrCl_2_(btd) is a quasi-two-dimensional *S* = 2 antiferromagnet with small single-ion anisotropy. Superexchange through the btd ligand in CrCl_2_(btd) is sixfold larger and of the opposite sign to superexchange through the pym ligand of CrCl_2_(pym). Density-functional theory (DFT) and cyclic voltammetry reveal that this strong superexchange arises from a reduction in Δ_CT_, demonstrating how ligand design can be used to modulate superexchange energy and magnetic dimensionality. This suggests that organic chemical functionalization to modulate the redox properties of ligands can be used to improve the magnetic function of MOFs without the formation of ligand radicals.

## Results

### Synthesis and Crystal Structure

We synthesized CrCl_2_(btd) by the reaction of CrCl_2_ and btd in tetrahydrofuran (THF) under reflux. Unlike the analogous MCl_2_(btd) with late transition metals (M = Fe, Co, Ni, Cu), phase-pure microcrystalline CrCl_2_(btd) does not form from solution under ambient conditions^2,7,21,22^ or by heating in neat combination.^2^

Having confirmed from PXRD that CrCl_2_(btd) has a MCl_2_(btd)-type structure (Figure S1), a high quality structural model was determined by Rietveld refinement against PND data of the deuterated analogue CrCl_2_(btd*-d*_4_) measured on the WISH diffractometer at 60 K.^26^ We used as a starting point a model generated through DFT-optimization of an atom-swapped NiCl_2_(btd) (Figure 1). The unit cell parameters and Cl atomic coordinates were allowed to refine freely; the btd molecule was refined as a rigid body, and the Cr atomic coordinates were fixed as they lie on special positions. CrCl_2_(btd) crystallizes in the monoclinic space group *P*2_1_/*m* with two formula units in the unit cell, like the Ni, Fe, and Co analogues (Table 1).^2,22^ The Cr^2+^ are coordinated by four Cl^–^ ligands and two N atoms from the btd ligands, which form a distorted CrCl_4_N_2_ octahedron (Figure 1c). The chromium octahedra edge-share through the Cl^–^ ligands along the crystallographic *a*-direction, and these chains are connected by btd alternating between upward in the *c*-direction and downward in the *c*-direction along the *b*-axis (Figure 1a,b). These layers stack in the crystallographic *c*-direction through vdW interactions (Figure 1b). The Cr^2+^ ion has a large JT distortion, with a long Cr–Cl bond, *d*_Cr–Cl_ = 2.753(4) Å, (c.f. CrCl_2_(pym), *d*_Cr–Cl_ = 2.761(5) Å, Cr^2+^Cl_2_(pyridine)_4_, *d*_Cr–Cl_ = 2.803(1) Å) and a short Cr–Cl, *d*_Cr–Cl_ = 2.338(3) Å, (c.f. CrCl_2_(pym), *d*_Cr–Cl_ = 2.3952(4) Å).^7,27^

**Figure 1:**
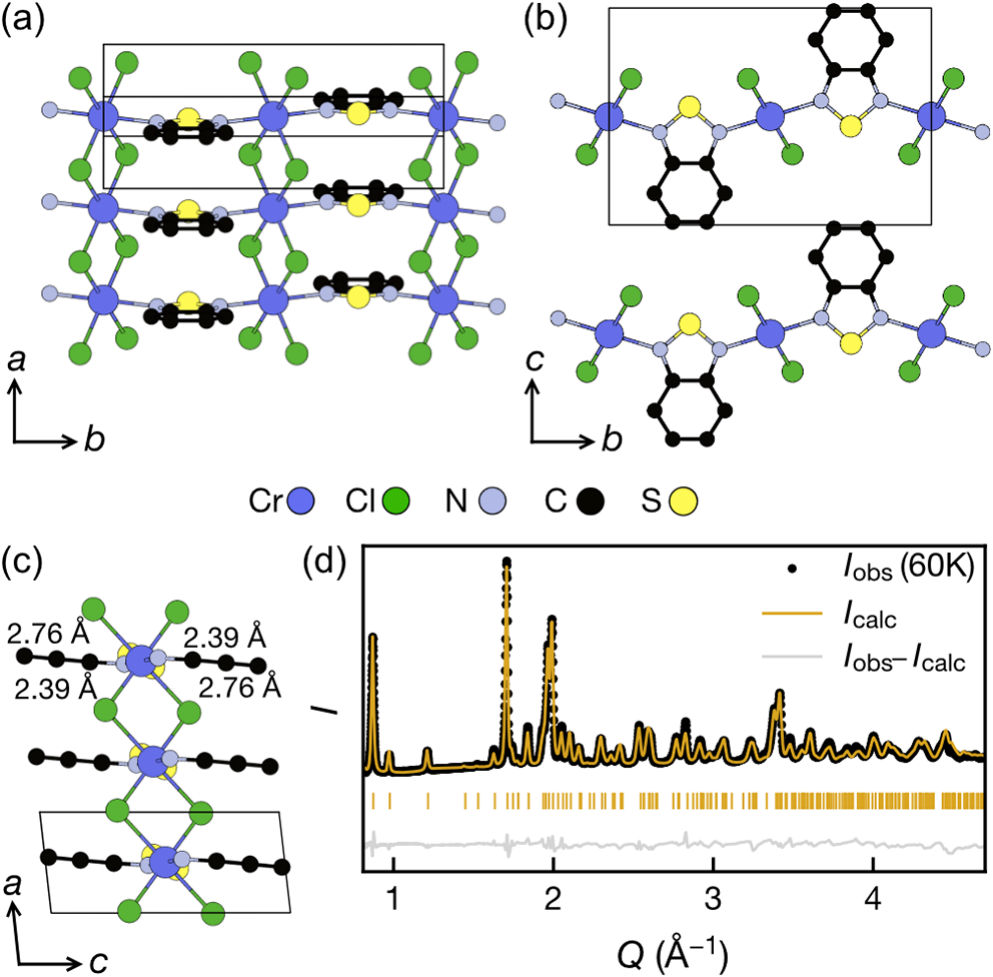
Crystal structure of CrCl_2_(btd) viewed along the (a) *c*, (b) *a*, and (c) *b* axes. Cr–Cl bond lengths are labeled, and H atoms are omitted for clarity. (d) Rietveld refinement^24^ of powder neutron diffraction data collected at 60 K of CrCl_2_(btd*-d*_4_) from bank 3 of the WISH instrument at the ISIS pulsed neutron and muon source.^25^

**Table 1:** Refined Lattice Parameters from PND Analysis of the Neutron Diffraction Pattern at 60 K

CrCl_2_(btd*-d*_4_)	
MW (g mol^–1^)	263.1
crystal system	monoclinic
space group	*P*2_1_/*m*
*a* (Å)	3.69759(6)
*b* (Å)	12.8982(6)
*c* (Å)	8.7554(4)
β (°)	96.741(4)
*V* (Å^3^)	414.68(3)
*T* (K)	60
*Z*	2
*R*_wp_	4.422
GOF	0.079

bond lengths, *r* (Å)	
Cr–Cl1	2.338(3)
Cr–Cl2	2.753(4)
Cr–N	2.0798(12)

Using single-crystal electron diffraction (SCED), we were able to determine the structure of nanocrystallites within the sample, which produced the same *P*2_1_/*m* structure found from neutron diffraction in multiple high-quality data sets. However, we also found two crystallites that had both superlattice reflections indicative of doubling along the *c-*axis and substantial twinning. Despite the presence of twinning, we were able to solve the structure of these crystallites, which revealed a very subtle rotation in btd ligands and CrCl_2_ chains around the *a-axis*, along with differences in the atomic displacement parameters compared to the bulk structure, which alternate along the *c-*axis. We find no evidence for a doubled *c-axis* in our powder diffraction data sets, and refinement of a multiphase model produces a refined phase fraction of ca. 1% doubled *c* phase, which is an upper limit due to the strong structural similarity. It is thus a negligible component of the bulk sample (Section S2, Table S1).

### Magnetic Susceptibility

We investigated the magnetic properties of CrCl_2_(btd*-d*_4_) by first measuring the temperature-dependent magnetic susceptibility, χ­(*T*), under field cooled (FC) and zero-field cooled (ZFC) conditions in a 0.01 T *dc* field from 2 to 300 K. The susceptibility has a broad peak at 60 K, characteristic of low-dimensional short-range order (Figure 2a) and there is a discontinuity in the temperature derivative, $\frac{d\chi }{\mathrm{dT}}\left(T\right)$ , at *T*_N_ = 46(2) K, indicative of antiferromagnetic order (Figure 2d).^29,30^ The nearest-neighbor magnetic Hamiltonian

1 $$H=\sum_{\left\langle \mathrm{ij}\right\rangle }-J_{\mathrm{ij}}S_{i}\cdot S_{j}+\sum_{i}D{\left(S_{i}^{z}\right)}^{2}$$

**Figure 2:**
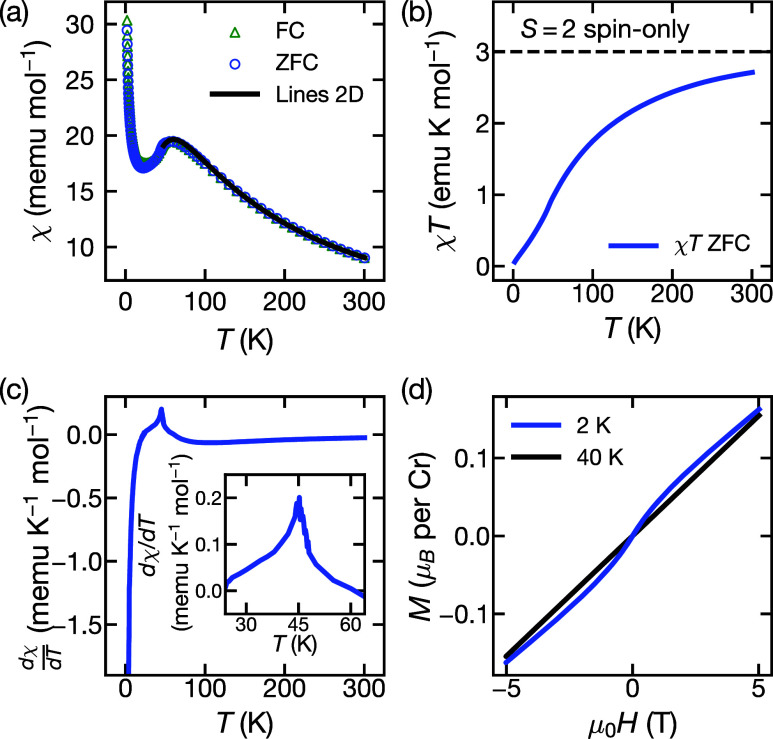
Magnetic susceptibility, χ, measurements of CrCl_2_(btd*-d*_4_). (a) χ­(*T*) measured under zero-field cooled (ZFC) and field cooled (FC) conditions in a 0.01 T *dc* field, with a 2D Lines’ model^28^ fit carried out over *T* > 48 K. (b) *χT*(*T*) in ZFC conditions 2–300 K. Dashed line shows the *S* = 2 spin-only limit. (c) ZFC $\frac{d\chi }{\mathrm{dT}}\left(T\right)$ over 2–300 K. Inset: $\frac{d\chi }{\mathrm{dT}}\left(T\right)$ over 24–64 K. (d) Isothermal magnetization, *M*(μ_0_H) measured at 2 and 40 K between −5 to 5 T.

comprising Heisenberg exchange, *J*_*ij*_, for the three nearest neighbors (that is, along the CrCl_2_ *a*, through the btd ligand *b*, and between vdW layers *c*) and single-ion anisotropy, *D* (Figure 3a,b), is a good first approximation for the key magnetic properties of this system. However, we explored fitting the susceptibility data (*T* > 48 K, outside the vicinity of the ordering transition) with two minimal models, the one-dimensional (1D) Heisenberg AFM Fisher model, with only one superexchange parameter being nonzero *J*_Cl_ or *J*_btd_ (these two alternatives cannot be distinguished in powder susceptibility data),^31^ and the 2D quadratic Heisenberg AFM Lines’ model (*J*_Cl_ = *J*_btd_ = *J*_2D_ ≠ 0) (Figure 2c),^28^ assuming Heisenberg anisotropy (*D* = 0) and negligible interlayer interactions (*J*_vdW_ = 0). The 2D square Lines’ model fitted the data better, *g*_2D_ = 2.15(10) and *J*_2D_ = 9.6(5) K, suggesting that CrCl_2_(btd) is closer to a 2D AFM over the temperature range 48 < *T* < 300 K.

**Figure 3:**
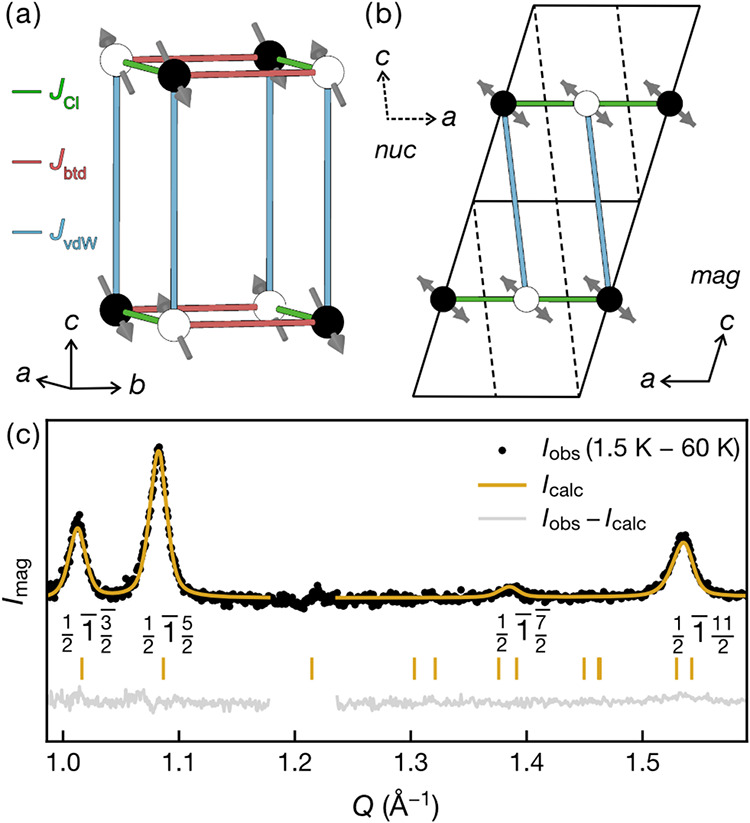
(a) The magnetic structure of CrCl_2_(btd*-d*_4_), highlighting the three largest exchange interactions, *J*_Cl_, *J*_btd_, and *J*_vdW_. Only one nuclear cell is shown. (b) The magnetic structure of CrCl_2_(btd*-d*_4_) viewed along the *b*-axis showing the magnetic unit cell (solid) and nuclear unit cell (dashed). (c) Rietveld refinement of temperature-subtracted neutron scattering data collected from the WISH instrument bank 2. The magnetic Bragg peaks are indexed relative to the parent nuclear cell.

At low temperatures (*T* < 10 K), there is a steep rise in susceptibility, which we were able to fit by 1.1(3) spin % of isolated paramagnetic spins. This suggests either domain boundaries or a small fraction of Cr^3+^ formed through surface oxidation.

The isothermal magnetization, *M*(*H*) at *T* = 2 and 40 K, shows no evidence of saturation at μ_0_H = 5 T, consistent with the fitted antiferromagnetic interactions (Figure 2). In addition to the expected gradual linear increase in *M*(*H*), we observe at *T* = 2 K an initial rapid rise, which saturates by approximately 1 T. This can be fitted by including a small concentration of a paramagnetic impurity (0.8(2)%) obeying the Brillouin-function dependence in addition to the linear antiferromagnetic bulk.

### Magnetic Ground State

We investigated the magnetic ground state using variable-temperature powder neutron diffraction patterns measured with the WISH diffractometer at the ISIS Neutron and Muon Source.^25^ We isolated the magnetic contribution from the nuclear structural diffraction by subtracting a data set above *T*_N_ (60 K) from the lowest temperature data set (1.5 K), which revealed four clear magnetic Bragg peaks. We were able to index these peaks using the propagation vector $k=\left(\frac{1}{2},0,\frac{1}{2}\right)$ , and using the ISODISTORT software, we identified the two possible irreducible representations (irreps), *mA*_1_^+^ and *mA*_2_^+^.^32^ We carried out Rietveld refinement of the magnetic structures produced by each irrep, which showed that only the *mA*_1_^+^ irrep was consistent with the experimental data (Figure 3, Table 2).

**Table 2:** Magnetic Structure Refined against PND Data^a^

CrCl_2_(btd)	
crystal system	monoclinic
magnetic space group	*P*2_1_/*m*.1_*a*_^′^ (UNI)^33^
**k**-vector	$\frac{1}{2}\;0\;\frac{1}{2}$
*M*_*x*_ (μ_B_)	2.21(3)^a^
*M*_*y*_ (μ_B_)	0.000(7)^a^ ^,^ ^b^
*M*_*z*_ (μ_B_)	1.93(2)^a^
*M*_0_ (μ_B_)	2.94(2)
$C_{\mathrm{Cl}}$	AFM
$C_{\mathrm{btd}}$	AFM
$C_{\mathrm{vdW}}$	AFM
*T* (K)	1.5
*R*_wp_	12.084
GOF	0.010

a Ordered moment specified in Cartesian basis, *x* = *a*_nuc_, *y* = *b*_nuc_, *z* = *c*_nuc_ × sin *β*.

b The *b* component was fixed to zero as the relevant Bragg peak was not observed. Uncertainty was derived from freely refining this parameter using a starting value of zero.

The magnetic structure resulting from this refinement is collinear, with antiferromagnetically correlated CrCl_2_ spin chains in turn antiferromagnetically correlated through the btd ligands. These antiferromagnetic layers are themselves correlated antiferromagnetically (Figure 3a and b). The refined Cr magnetic moment was *M*_0_ = 2.94(2) μ_B_, significantly less than the spin-only value of M = *gS* = 4 μ_B_. This is consistent with the significant spin delocalization observed in our DFT calculations (vide infra). The fitted moment could also be reduced by the presence of magnetic disorder due to magnetic stacking faults, though no clear indication of a reduced correlation length was found in the magnetic peak widths. The magnetic space group is *P*2_1_/*m*.1_*a*_^′^ (UNI).^33^ The magnetic unit cell is related to the nuclear cell by the following transformation matrix

$$\left(\begin{array}{l} a_{\mathrm{mag}}\\ b_{\mathrm{mag}}\\ c_{\mathrm{mag}} \end{array}\right)=\left(\begin{array}{rrr} 2 & 0 & 0\\ 0 & -1 & 0\\ -1 & 0 & -1 \end{array}\right)\cdot \left(\begin{array}{l} a_{\mathrm{nuc}}\\ b_{\mathrm{nuc}}\\ c_{\mathrm{nuc}} \end{array}\right)$$

The orientation of the magnetic moments could be robustly refined, and we found that they lie within the *ac*-plane, lying broadly along the Jahn–Teller axis, i.e., the long Cr–Cl bonds. A component of the moment lying along the *b*-direction—which would produce noncollinearity—is permitted by symmetry. The presence of a component along *b* would result in intensity at the $\frac{1}{2}0{\frac{1}{2}}_{\mathrm{nuc}}$ peak position (*Q* = 0.96 Å^–1^), which is not seen in our data, so any noncollinearity is negligible with an angular deviation from collinearity θ < 0.2° (Table 2).

### Magnetic Interactions

Our magnetic susceptibility data suggest that superexchange is significantly enhanced in CrCl_2_(btd) compared to other vdW MOMs.^2,7,34^ To confirm this, and in particular whether *J*_btd_ or *J*_Cl_ is responsible for this increase, we measured INS data. We measured the same powder sample of CrCl_2_(btd*-d*_4_) at 1.7, 60, and 285 K using the LET spectrometer at ISIS, using rep-rate multiplication to measure at multiple *E*_*i*_ simultaneously (*E*_*i*_ = 12.14, 3.70, 1.77 meV).^35^ The spectra collected at 1.7 K show clear magnetic excitations with the maximum intensity centered at Δ*E* = 8.0(3) meV and an energy gap of 3.0(1) meV (Figure 4).

**Figure 4:**
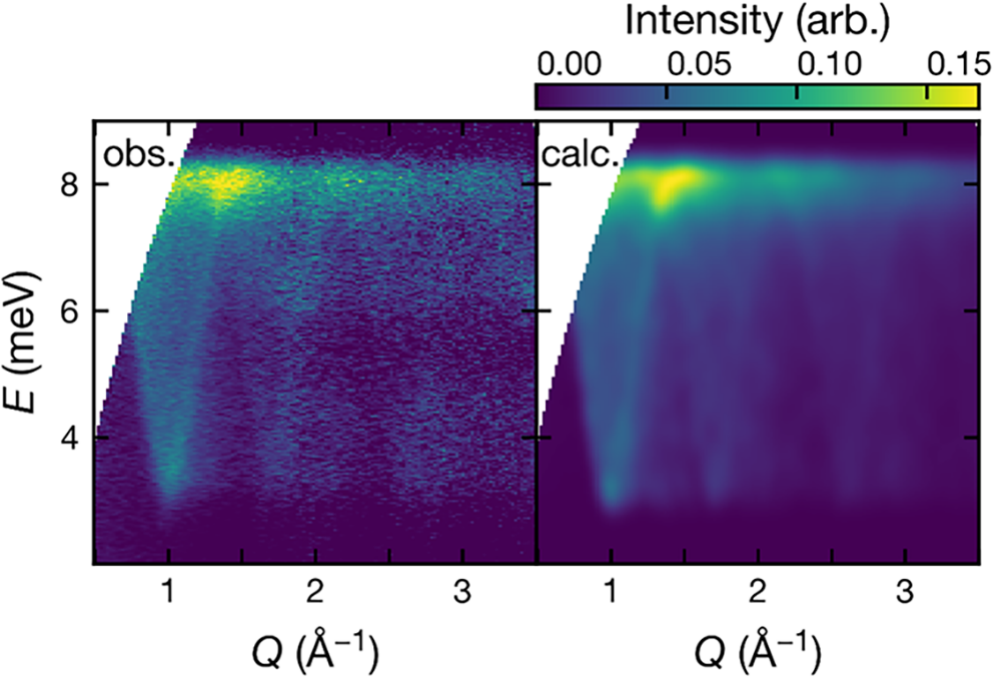
Time-of-flight powder INS spectra of CrCl_2_(btd*-d*_4_) with *E*_*i*_ = 12.14 meV measured at 1.7 K with a fitted background subtracted (obs). LSWT-calculated scattering intensity fitted to the 1.7 K data, with parameters *J*_Cl_ = −15.31(84), *J*_btd_ = −6.96(54), *J*_vdW_ = −0.1(1), and *D* = −2.0(2) K with a background linear in *Q* and linear in *E* (calc.). The Hamiltonian is described in eq 1.

The intensity of these features rapidly decreases with increasing *Q*, until they are masked by phonons, indicating their magnetic origin (Figures 3 and S18). We were able to quantitatively fit these data using linear spin wave theory (LSWT)^36^ as implemented in the SpinW software package using the full magnetic Hamiltonian (eq 1; Figure 3).

We began by estimating the approximate values for each of *J*_Cl_, *J*_btd_, *J*_vdW_, and *D* using the bulk magnetic measurements and the values determined for CrCl_2_(pym). These initial parameters were then improved using iterative minimization of the least-squares discrepancy between experimental and calculated spectra, including a refined multiplicative scale factor and a background linear in both *Q* and Δ*E*, over the range 0.5 < *Q* < 3.5 Å^–1^ and 2 < Δ*E* < 9 meV. This first fitting gave *J*_Cl_ + *J*_btd_ = −22.1(1.1) K, 0 > *J*_vdW_ > −0.1(1) K, and *D* = −2.0(2) K. All reported anisotropies *D* have been corrected for kinematical consistency,^37^ as by default SpinW uses the inconsistent $D^{\prime }=D\left[1-\frac{1}{2S}\right]=\frac{3}{4}D$ .

To deconvolve *J*_Cl_ and *J*_btd_, we carried out a coarse grid search varying these two *J* parameters (Δ­(*J*) = 1 K), confirming that the line in parameter space *J*_Cl_ + *J*_btd_ = −22.1(1.1) K gives the best fit. The global minimum in the fit along this line was at *J*_Cl_ = −15.1(1.2) K and *J*_btd_ = −6.96(1.2) K, with an additional local minimum occurring where *J*_Cl_ and *J*_btd_ interchanged (Figure S17a,c). Visual inspection confirmed that the global minimum better accounted for the experimental data in addition to having the lower residual χ^2^ (Figure S16). A finer grid search (Δ­(*J*) = 0.2 K) over approximately −16 > *J*_Cl_ > −14 K and −8 > *J*_btd_ > −0.6 K localized this minimum at *J*_Cl_ = −15.31(84) K and *J*_btd_ = −6.96(54) K (Figures 4 & S17b).

This corresponds to an average 2D intralayer exchange $\frac{1}{2}\left(J_{\mathrm{Cl}}+J_{\mathrm{btd}}\right)=11.1\left(6\right)$ K, which is consistent with our magnetic susceptibility data, *J*_2D_ = 9.6(5) K (Figure 2c). *J*_vdW_ was zero within error in our data; however, the ground state determined by PND indicates that *J*_vdW_ must be antiferromagnetic.

### Electronic Structure

To understand the origin of the enhancement of exchange, we therefore turned to first-principles spin-polarized density-functional theory (DFT), comparing these results directly to our previous work on CrCl_2_(pym).^38–40^ We relaxed the primitive cell of CrCl_2_(btd), starting from the experimental model, using a Hubbard *U*_eff_ correction to account for highly localized Cr d-orbitals and an MBD* many-body dispersion correction. We explored a range of *U*_eff_ values and found that *U*_eff_ = 3 eV produced the structure and electronic density most consistent with both experiment and geometry optimization calculations carried out using higher-level hybrid functionals (HSE06) (Section S3; Table S2). This is the *U*_eff_ we found optimal for our previous calculations of CrCl_2_(pym). Lower values of *U*_eff_ ≤ 1 eV do not have the observed large Jahn–Teller distortion, suggesting overly delocalized d-electrons.

Having identified an appropriate parametrization, we therefore calculated Heisenberg exchange parameters using a broken symmetry approach, fitting the magnetic Heisenberg spin Hamiltonian (i.e., eq 1 where *D* = 0) to the energies of the eight distinct 2 × 1 × 2 supercells constructed from the relaxed primitive cell (containing two Cr^2+^ ions), where the Cr spins are varied between spin-up and spin-down orientations (Table S3).^41^ We found that the hierarchy of exchange interactions was robust to changes in *U*_eff_ and that the exchange energies predictably decreased in magnitude with increasing *U*_eff_ and hence d-orbital localization (Table S3).

Our calculations qualitatively reproduced those determined by experiment, with antiferromagnetic *J* along all three considered superexchange pathways and *J*_btd_ ≈ *J*_Cl_ ≫ *J*_vdW_, though our calculations erroneously predict *J*_btd_ to be slightly larger than *J*_Cl_, rather than the reverse, which is experimentally observed. Examination of the spin density suggests that the spin exchange pathway lies primarily along the Cr–N–C–C–N–Cr route in CrCl_2_(btd) (i.e., five-bond superexchange), rather than Cr–N–S–N–Cr (i.e., four-bond superexchange) (Figures 5(a,b),S5,S6). The electronic density of states produced using hybrid functionals shows that the band gap is significantly reduced from CrCl_2_(pym) (*E*_*g*_ = 2.19 eV) to CrCl_2_(btd) (*E*_*g*_ = 1.51 eV) (Figures S11, S13). Examination of the full band structure produced using DFT+*U* showed the same trends for all values of *U*_eff_ (Table S4), and demonstrated that the change in *E*_*g*_ primarily results from the lower energy LUMO of the btd ligand, rather than changes in the bandwidth of the ligand-derived bands, which π-stack along the a-axis, which are similar in both CrCl_2_(btd) and CrCl_2_(pym) (Figures 5,S11–S14 and Table S5).

**Figure 5:**
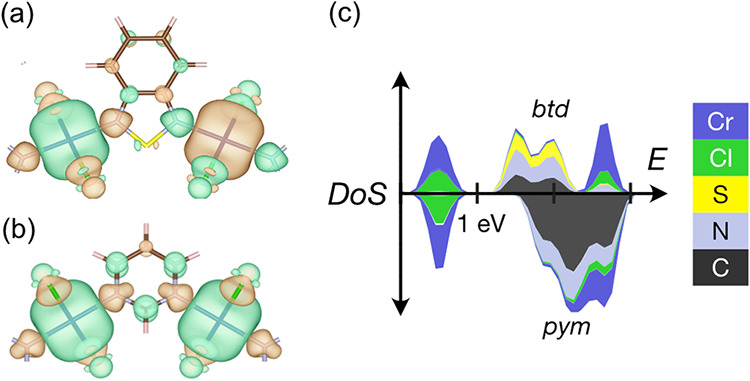
Result of DFT+*U* calculations (*U*_eff_ = 3 eV). Spin density isosurfaces for (a) CrCl_2_(btd) and (b) CrCl_2_(pym). (c) Electronic density of states (DoS) partitioned by element, with CrCl_2_(btd) shown on the top and CrCl_2_(pym) on the bottom.

We also directly measured the redox potentials of the ligands using solution cyclic voltammetry measurements, which showed btd to exhibit a reduction onset potential of *E*_onset_ = −1.55 V versus ferrocenium|ferrocene, a +0.62 V positive shift compared to the reduction onset potential of pym (*E*_onset_ = −2.17 V) (Figures S19–S21). This positive shift of 0.62 V is similar to the change in charge-transfer gap calculated using hybrid DFT, Δ*E*_g_ = 0.68 eV.

## Discussion

The structure of CrCl_2_(btd) is entirely consistent with MCl_2_L metal–organic magnets: the Jahn–Teller distortion is similar in size to CrCl_2_(pym),^7^ the ligand geometry is the same as MCl_2_(btd),^2,21,22^ and even the orbital ordering pattern is identical to that of CuCl_2_(btd).^21^ We also find that the direction of the moments (approximately along the Jahn–Teller axis) is consistent between CrCl_2_(btd) and CrCl_2_(pym),^7^ the expected easy axis of magnetic anisotropy for an octahedral d^4^ metal.^42^

However, our electron diffraction results uncover hidden polymorphism, particularly associated with stacking disorder, which was not detected in powder X-ray and neutron measurements. This is likely to be a common feature of powder samples^43^ and should be considered carefully for any measurement particularly sensitive to low-concentration impurities, e.g., catalysis. However, magnetic measurements are robust to these low-concentration impurities, particularly as the differences in the magnetic Hamiltonian between these polymorphs are likely to be very small.

Although the structure of CrCl_2_(btd) is consistent with previous structures, we find that CrCl_2_(btd) stands separately in its magnetic function. In our and others’ previous work, it has been found that the differences in magnetic function between MCl_2_(pym) and MCl_2_(btd) are small, for example NiCl_2_(btd) *T*_*C*_ = 17.5(5) K and NiCl_2_(pym) *T*_*C*_ = 15.8(7) K.^2^ This similarity is not surprising, as the chloride chains are largely electronically and geometrically similar between the pairs of compounds so *J*_Cl_ is expected to be largely unchanged, and *J*_Cl_ will also dominate the resultant *T*_*C*_.

This is not true for Cr^2+^. Our INS measurements show that *J*_btd_ = −6.96(54) K in contrast to *J*_pym_ = +1.2(2) K, an increase in magnitude by a factor of 6, while *J*_Cl_ increases only by about a sixth: *J*_Cl_ = −15.3(84) K for CrCl_2_(btd) and *J*_Cl_ = −13.1(5) K for CrCl_2_(pym). This significant enhancement in superexchange through a N-heterocyclic ligand has been seen in reducing VCl_2_(pyrazine)_2_,^13^ for which *J* = −28.2(5) K is orders of magnitude stronger than the late transition metal analogues (e.g., NiCl_2_(pyrazine)_2_ *J* = −0.49(1) K).^6^ The large enhancement in *J* is likely the smaller charge-transfer gap, as *J* ∝ Δ_CT_^–3^ for a charge-transfer insulator.^44^ VCl_2_(pyz)_2_ and CrCl_2_(btd) are two distinct routes for reducing Δ_CT_: increasing the metal HOMO energy, VCl_2_(pyz)_2_, and decreasing the ligand LUMO energy, CrCl_2_(btd) (Figure 6). Fine-tuning of the metal HOMO level has been previously achieved through the ligand field control in CrX_2_(pyz)_2_, where a valence-tautomeric switch from positive to negative Δ_CT_ can be achieved through replacing I by Br in solid solutions.^4^ Although Δ_CT_ is likely the controlling factor for the enhancement of *J*, the more diffuse metal d-orbitals likely also contribute to the stronger exchange in these early transition metal complexes.

**Figure 6:**
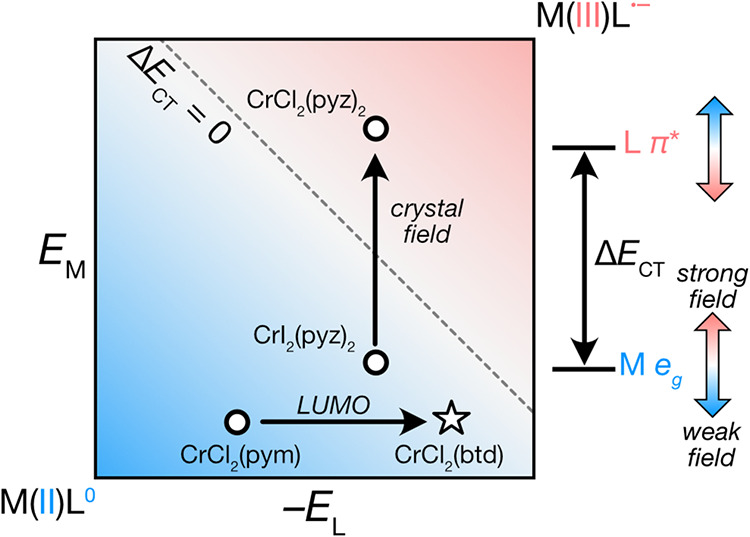
Schematic illustration of the charge-transfer gap tuning, Δ_CT_. Increasing the ligand field strength from I^–^ to Cl^–^ increases the energy of the Cr *e*_*g*_ electron, the HOMO, and hence decreases Δ_CT_, in fact making Δ_CT_ < 0 for CrCl_2_(pyz)_2_. Reducing the organic ligand LUMO energy from pym to btd also decreases Δ_CT_.

Although the shortest through-bond pathway connecting the Cr atoms through the organic ligands is four bonds for both CrCl_2_(pym) and CrCl_2_(btd) (Cr–N–X–N–Cr), our DFT calculations suggest that, in fact, the longer five-bond pathway (Cr–N–C–C–N–Cr) is more important for CrCl_2_(btd). As the sign of the spin density typically alternates along the exchange pathway,^45^ this could explain the antiferromagnetic *J*_btd_ and ferromagnetic *J*_pym_.

A detailed understanding of the local interactions is essential for determining which chemical features underlie the observed magnetic properties. INS has proven to be an invaluable tool for probing these local interactions in early transition metal–organic magnets.^4,7^ In particular, we found that careful analysis of powder INS data was able to disentangle *J*_Cl_ from *J*_btd_, which would otherwise be challenging to independently determine. This ability to deconvolute distinct superexchange pathways is one of the key advantages of INS.^46,47^

## Conclusion

We have determined the crystal structure, bulk magnetic properties, magnetic ground state, and magnetic excitations of a coordination polymer, CrCl_2_(btd). We have shown that this compound contains Cr^2+^ despite the low-energy LUMO of the btd linker, in contrast to other CrCl_2_L_*x*_ MOMs, which contain Cr^3+^-radical ligand pairs.^48,49^ Nonetheless, the redox-active btd ligand enhances the superexchange compared to pyrimidine, with *J*_*L*_ increasing by a factor of six, likely as a result of its smaller charge-transfer gap. Engineering of the frontier orbital energies has the potential to significantly enhance magnetic interactions in MOMs containing diamagnetic ligands. In particular, we demonstrate that control over the organic ligand orbital energies can be used to generate large changes in the magnetic function.

## Methods

### Synthesis

Synthesis and handling of CrCl_2_(btd) were performed in a dry Ar or N_2_ atmosphere using an MBraun LABstar glovebox or Schlenk line. The reaction of CrCl_2_ (474 mg, 3.85 mmol; Fisher Scientific, 99.9%) and 2,1,3-benzothiadiazole (579 mg, 4.25 mmol; Acros Organics, 98.0%) in 25 mL THF at 120 °C under reflux with stirring for 1 day precipitated a green-brown microcrystalline powder. The CrCl_2_(btd) product was filtered and dried *in vacuo* (80% yield). This procedure, using 2,1,3-benzothiadiazole-*d*_4_ and the quantities scaled up, was used to produce samples for neutron scattering studies in three batches: CrCl_2_ (474 mg, 3.85 mmol; 356 mg, 2.90 mmol; 185 mg, 1.51 mmol) and 2,1,3-benzothiadiazole-*d*_4_ (600 mg, 4.25 mmol; 447 mg, 3.28 mmol; 225 mg, 1.65 mmol). The synthesis of btd*-d*_4_ was described previously.^2^

### Powder X-ray Diffraction

PXRD data were collected using a PANalytical X’Pert Pro diffractometer equipped with monochromated Cu Kα_1_ radiation (λ = 1.5406 Å). The tube voltage and current were 40 kV and 40 mA, respectively. Scans were performed from 2° to 80° on a zero background silicon crystal plate. Peak fitting, Pawley, and Rietveld refinement were performed using Topas Academic v6.^26^

### Electron Diffraction

A portion of CrCl_2_(btd) was dispersed dry onto a copper-supported holey amorphous carbon TEM grid and loaded at room temperature via a single-tilt high-tilt JEOL specimen holder into a Rigaku XtaLAB Synergy-ED electron diffractometer, operated at 200 kV and equipped with a Rigaku HyPix-ED hybrid pixel array area detector. Data were collected as single-rotation scans collecting 0.25° frames using the CrysAlisPRO system (CCD 1.171.43.95a 64-bit release 03–11–2023) using continuous rotation electron diffraction with a selected area aperture of 2 μm apparent diameter.^50^ All single crystal component data sets were individually indexed and integrated, prior to merging of suitable data sets of each phase for scaling using CrysAlisPRO (version 1.171.44.112a);^50^ no absorption corrections were applied. The structures were solved using ShelXT^51^ and refined using Olex2.refine in the kinematic approximation, applying extinction corrections to broadly account for multiple scattering alongside judicious rejection of outlying reflections, as implemented in Olex2 (version 1.5-ac7–014, compiled 2025.02.27 svn.r6f4c0eaf for Rigaku Oxford Diffraction, GUI svn.r7171),^52,53^ using published scattering factors.^54^ Precise experimental details are provided in Table S1 and Section S2.

### Magnetic Susceptibility

Magnetic property measurements were carried out on a Quantum Design MPMS-XL. A polycrystalline sample of CrCl_2_(btd*-d*_4_) (10.7 mg) was immobilized in eicosane (19.0 mg) and sealed in a low-paramagnetic-impurity borosilicate glass ampule under vacuum. Magnetic susceptibility measurements were performed under field cooled (FC) and zero-field cooled (ZFC) conditions in a 0.01 T *dc* field from 2 to 300 K. Isothermal magnetization measurements were performed at 2 K from 0 to 5 T to −5 to 5 T. Data were corrected for the diamagnetism of the sample using Pascal’s constants.^55^

### Powder Neutron Diffraction

Powder neutron diffraction measurements were carried out with a sample of CrCl_2_(btd*-d*_4_) (1.3 g) on the WISH instrument at the ISIS Neutron and Muon Source.^25^ The nuclear structure was determined by Rietveld refinement against neutron diffraction data collected at 60 K from banks 2 and 3. Due to the low intensity of magnetic reflections, the magnetic structure was determined by refinement against data from which background and nuclear Bragg peaks were removed by subtraction of data collected at 60 K from those collected at 1.5 K. The magnetic Bragg peaks were indexed to determine the magnetic propagation vector, and then the allowed magnetic irreducible representations were determined using symmetry-mode analysis on the ISODISTORT software.^56^ Using the scale factor determined from Rietveld refinement of the nuclear structure against data at 60 K, and peak parameters determined from Rietveld refinement of a NaCaAlF_6_ calibrant, the direction and magnitude of the ordered moment for the subtracted data set were refined using TOPAS-ACADEMIC 6.0.^26^

### Inelastic Neutron Scattering

Inelastic neutron scattering (INS) measurements were performed on the LET time-of-flight direct-geometry spectrometer at ISIS.^35^ The sample (1.3 g) was contained in a thin annular aluminum can with a diameter of 15 mm, an annular width of 1 mm, and a height of 45 mm, and was cooled in a helium cryostat. The data were collected at 1.7 and 60 K for 5 h each and at 285 K for 1 h, with *E*_*i*_ = 12.14 meV using the rep-rate multiplication method.^57,58^ The data were reduced using the Mantid-Plot software package.^59^ The raw data were corrected for detector efficiency and time-independent background following standard procedures.^60^

### Density-Functional Theory

The plane-wave DFT code CASTEP version 23.1 was used for all calculations.^61^ The PBE generalized gradient approximation (GGA) functional was used together with norm-conserving pseudopotentials from the built-in NCP19 library.^62^ A cutoff energy of 1500 eV was used for the plane-wave basis set. van der Waals forces between each layer were treated using the MBD* dispersion correction.^63,64^ An effective on-site Hubbard *U* term, *U*_eff_ = *U*–*J*, was needed to impose a strong correlation on the Cr d-states, where *U* is the on-site Coulomb term and *J* is the site exchange term. The convergence criteria used for electronic self-consistency were 10^–10^ eV. A Monkhorst–Pack (MP) grid with *k*-point spacing of 2π × 0.03 Å^–1^ was used for the Brillouin zone integration.^65^ The LBFGS optimization algorithm was used for structural relaxations with a force tolerance of 0.03 eV Å^–1^.^66,67^

Hybrid functional calculations used the HSE06 functional with NCP19 norm-conserving pseudopotentials.^68^ The same cutoff energy and convergence criteria as used in the PBE calculations were used. An MP grid with *k*-point spacing of 2π × 0.08 Å^–1^ was used for the self-consistent cycle, and a finer MP grid with *k*-point spacing of 2π × 0.07 Å^–1^ was used for the density of states.

The OptaDOS code, together with the matador high-throughput environment, was used to generate band structures and densities of states.^69–72^ The c2x code was used to visualize spin density and Kohn–Sham orbitals.^73^ The effective-mass calculator by Fonari and Sutton, included in CASTEP utilities, was used.^74^

### Cyclic Voltammetry

All voltammetry experiments were carried out in a nitrogen-filled glovebox. MeCN was dried over molecular sieves until the water content was <10 ppm (checked with Karl Fischer titration). Voltammetry was carried out in 1 M LiTFSI dissolved in MeCN with an analyte concentration of 1 mM. A glassy carbon working electrode, platinum counter electrode, and silver wire pseudoreference were used. A scan rate of 100 mV s^–1^ was used. Ferrocene was added to the electrolyte post measurement, and its redox potential was recorded as an internal reference.

## Supplementary Material





## Notes

Additional powder X-ray diffraction data; details of electron crystallography; details of DFT calculations; and additional inelastic neutron scattering data (PDF)
Magnetic structure (mCIF); additional research data for this article may be accessed at no charge and under CC-BY license at the University of Nottingham Research Data Management Repository DOI: 10.17639/nott.7573 Neutron scattering data may be accessed at DOI: 10.5286/ISIS.E.RB2210345 and 10.5286/ISIS.E.RB2210360; CCDC 2457667, 2457668, and 2465690 contain the supporting crystallographic data for this paper; these data can be obtained free of charge via www.ccdc.cam.ac.uk/data_request/cif or by emailing data_request@ccdc.cam.ac.uk, or by contacting the Cambridge Crystallographic Data Centre, 12 Union Road, Cambridge CB2 1EZ, U.K.; fax: + 44 1223 336033 (ZIP)

## References

[1] Thorarinsdottir A. E., Harris T. D. (2020). Metal-Organic Framework Magnets. Chem. Rev..

[2] Pitcairn J., Ongkiko M. A., Iliceto A., Speakman P. J., Calder S., Cochran M. J., Paddison J. A. M., Liu C., Argent S. P., Morris A. J., Cliffe M. J. (2024). Controlling Noncollinear Ferromagnetism
in van Der Waals Metal–Organic Magnets. J. Am. Chem. Soc..

[3] López-Cabrelles J., Mañas-Valero S., Vitórica-Yrezábal I. J., Šiškins M., Lee M., Steeneken P. G., van der Zant H. S. J., Mínguez Espallargas G., Coronado E. (2021). Chemical Design
and Magnetic Ordering in Thin Layers of 2D Metal–Organic Frameworks
(MOFs). J. Am. Chem. Soc..

[4] Aribot F., Voigt L., Dunstan M. A. (2025). Molecular Alloying Drives
Valence Change in a van der Waals Antiferromagnet. Chem.

[5] López-Cabrelles J., Miguel-Casañ E., Esteve-Rochina M., Andres-Garcia E., Vitorica-Yrzebal I., Calbo J., Espallargas G. M. (2022). Multivariate
Sodalite Zeolitic Imidazolate Frameworks: A Direct Solvent-Free Synthesis. Chem. Sci..

[6] Liu J., Goddard P. A., Singleton J. (2016). Antiferromagnetism in
a Family of *S* = 1 Square Lattice Coordination Polymers
NiX_2_(Pyz)_2_ (X = Cl, Br, I, NCS; Pyz = Pyrazine). Inorg. Chem..

[7] Pitcairn J., Iliceto A., Cañadillas-Delgado L., Fabelo O., Liu C., Balz C., Weilhard A., Argent S. P., Morris A. J., Cliffe M. J. (2023). Low-Dimensional
Metal–Organic Magnets as a Route
toward the *S* = 2 Haldane Phase. J. Am. Chem. Soc..

[8] Miller J. S., Epstein A. J. (1994). Organic and Organometallic Molecular Magnetic Materials—Designer
Magnets. Angew. Chem., Int. Ed..

[9] Perlepe P., Oyarzabal I., Mailman A. (2020). Metal-Organic Magnets
with Large Coercivity and Ordering Temperatures up to 242 °C. Science.

[10] Goren N., Das T. K., Brown N., Gilead S., Yochelis S., Gazit E., Naaman R., Paltiel Y. (2021). Metal Organic Spin
Transistor. Nano Lett..

[11] Murphy R. A., McCone K. C., Claassen R., Holmgren E., Johnston-Halperin E., Long J. R. (2025). Electrodeposition
of Magnonic V­(Tetracyanoethylene)_2_ Thin Films. J. Am. Chem. Soc..

[12] Espallargas G. M., Coronado E. (2018). Magnetic Functionalities in MOFs: From the Framework
to the Pore. Chem. Soc. Rev..

[13] Perlepe P., Oyarzabal I., Voigt L. (2022). From an Antiferromagnetic
Insulator to a Strongly Correlated Metal in Square-Lattice MCl_2_(Pyrazine)_2_ Coordination Solids. Nat. Commun..

[14] Dong R., Zhang Z., Tranca D. C., Zhou S., Wang M., Adler P., Liao Z., Liu F., Sun Y., Shi W., Zhang Z., Zschech E., Mannsfeld S. C. B., Felser C., Feng X. (2018). A Coronene-Based Semiconducting
Two-Dimensional
Metal-Organic Framework with Ferromagnetic Behavior. Nat. Commun..

[15] Yang C., Dong R., Wang M. (2019). A Semiconducting Layered
Metal-Organic Framework Magnet. Nat. Commun..

[16] Park J. G., Collins B. A., Darago L. E., Runčevski T., Ziebel M. E., Aubrey M. L., Jiang H. Z. H., Velasquez E., Green M. A., Goodpaster J. D., Long J. R. (2021). Magnetic Ordering
through Itinerant Ferromagnetism in a Metal-Organic Framework. Nat. Chem..

[17] Broholm C., Cava R. J., Kivelson S. A., Nocera D. G., Norman M. R., Senthil T. (2020). Quantum Spin Liquids. Science.

[18] Bai L., Feng W., Liu S., Šmejkal L., Mokrousov Y., Yao Y. (2024). Altermagnetism: Exploring New Frontiers
in Magnetism and Spintronics. Adv. Funct. Mater..

[19] Baltz V., Manchon A., Tsoi M., Moriyama T., Ono T., Tserkovnyak Y. (2018). Antiferromagnetic Spintronics. Rev. Mod. Phys..

[20] Ferlay S., Mallah T., Ouahès R., Veillet P., Verdaguer M. (1995). A Room-Temperature
Organometallic Magnet Based on Prussian Blue. Nature.

[21] Papaefstathiou G. S., Tsohos A., Raptopoulou C. P., Terzis A., Psycharis V., Gatteschi D., Perlepes S. P. (2001). Crystal Engineering: Stacking Interactions
Control the Crystal Structures of Benzothiadiazole (Btd) and Its Complexes
with Copper­(II) and Copper­(I) Chlorides. Cryst.
Growth Des..

[22] Papaefstathiou G. S., Perlepes S. P., Escuer A., Vicente R., Gantis A., Raptopoulou C. P., Tsohos A., Psycharis V., Terzis A., Bakalbassis E. G. (2001). Topological
Control in Two-Dimensional
Cobalt­(II) Coordination Polymers by *π*-*π* Stacking Interactions: Synthesis, Spectroscopic
Characterization, Crystal Structure, and Magnetic Properties. J. Solid State Chem..

[23] Duan W., Huang J., Kowalski J. A. (2017). “Wine-Dark Sea”
in an Organic Flow Battery: Storing Negative Charge in 2,1,3-Benzothiadiazole
Radicals Leads to Improved Cyclability. ACS
Energy Lett..

[24] Rietveld H. M. (1969). A Profile
Refinement Method for Nuclear and Magnetic Structures. J. Appl. Crystallogr..

[25] Chapon L. C., Manuel P., Radaelli P. G., Benson C., Perrott L., Ansell S., Rhodes N. J., Raspino D., Duxbury D., Spill E., Norris J. (2011). Wish: The New Powder
and Single Crystal
Magnetic Diffractometer on the Second Target Station. Neutron News.

[26] Coelho A. A. (2018). TOPAS and
TOPAS-Academic: An Optimization Program Integrating Computer Algebra
and Crystallographic Objects Written in C++. J. Appl. Crystallogr..

[27] Cotton F. A., Daniels L. M., Feng X., Maloney D. J., Murillo C. A., Zúñiga L. A. (1995). Experimental and
Theoretical Study
of a Paradigm Jahn-Teller Molecule, All-*Trans*-CrCl_2_(H_2_O)_2_(Pyridine)_2_, and the
Related *Trans*-CrCl_2_(Pyridine)_4_·Acetone. Inorg. Chim. Acta.

[28] Lines M. E. (1970). The Quadratic-Layer
Antiferromagnet. J. Phys. Chem. Solids.

[29] Cliffe M. J., Lee J., Paddison J. A. M., Schott S., Mukherjee P., Gaultois M. W., Manuel P., Sirringhaus H., Dutton S. E., Grey C. P. (2018). Low-Dimensional
Quantum Magnetism
in Cu­(NCS)_2_: A Molecular Framework Material. Phys. Rev. B.

[30] Vasiliev A., Volkova O., Zvereva E., Markina M. (2018). Milestones
of Low-D
Quantum Magnetism. npj Quantum Mater..

[31] Fisher M. E. (1964). Magnetism
in One-Dimensional Systems-The Heisenberg Model for Infinite Spin. Am. J. Phys..

[32] Cracknell, A. P. ; Davies, B. ; Miller, S. C. ; Love, W. F. Kronecker Product Tables; IFI/Plenum: New York, 1979; Vol. 1.

[33] Campbell B. J., Stokes H. T., Perez-Mato J. M., Rodríguez-Carvajal J. (2022). Introducing
a Unified Magnetic Space-Group Symbol. Acta
Crystallogr..

[34] Perlepe P., Oyarzabal I., Pedersen K. S., Negrier P., Mondieig D., Rouzières M., Hillard E. A., Wilhelm F., Rogalev A., Suturina E. A., Mathonière C., Clérac R. (2018). Cr­(Pyrazine)_2_(OSO_2_CH_3_)_2_: A Two-Dimensional
Coordination Polymer with an Antiferromagnetic Ground State. Polyhedron.

[35] Bewley R. I., Taylor J. W., Bennington S. M. (2011). LET, a
Cold Neutron Multi-Disk Chopper
Spectrometer at ISIS. Nucl. Instrum. Methods
Phys. Res..

[36] Toth S., Lake B. (2015). Linear Spin Wave Theory
for Single-Q Incommensurate Magnetic Structures. J. Phys.: Condens. Matter.

[37] Balucani U., Tognetti V., Pini M. G. (1979). Kinematic
Consistency in Anisotropic
Ferromagnets. J. Phys. C: Solid State Phys..

[38] Hohenberg P., Kohn W. (1964). Inhomogeneous electron
gas. Phys. Rev..

[39] Kohn W., Sham L. J. (1965). Self-consistent equations including
exchange and correlation
effects. Phys. Rev..

[40] Payne M. C., Teter M. P., Allan D. C., Arias T., Joannopoulos J. D. (1992). Iterative
minimization techniques for ab initio total-energy calculations -
molecular-dynamics and conjugate gradients. Rev. Mod. Phys..

[41] Ciofini I. (2003). DFT calculations
of molecular magnetic properties of coordination compounds. Coord. Chem. Rev..

[42] Oshio H., Nakano M. (2005). High-Spin Molecules with Magnetic
Anisotropy toward
Single-Molecule Magnets. Chem. - Eur. J..

[43] Myatt E., Lata S., Pitcairn J., Daisenberger D., M Kronawitter S., A Hallweger S., Kieslich G., P Argent S., P Tidey J., J Cliffe M. (2024). Ligand Solid-Solution Tuning of Magnetic
and Mechanical Properties of the van Der Waals Metal–Organic
Magnet NiCl_2_ (btd)_1–*x*
_(bod)_
*x*
_. Chem. Commun..

[44] Khomskii, D. I. Transition Metal Compounds; Cambridge University Press: Cambridge, 2014.

[45] Kahn, O. Molecular Magnetism; Dover Publications: Garden City, NY, 2021.

[46] Walker H. C., Duncan H. D., Le M. D., Keen D. A., Voneshen D. J., Phillips A. E. (2017). Magnetic Structure and Spin-Wave Excitations in the
Multiferroic Magnetic Metal-Organic Framework (CD_3_)_2_ND_2_[Mn­(DCO_2_)_3_]. Phys. Rev. B.

[47] Mole R. A., Greene S., Henry P. F., Humphrey S. M., Rule K. C., Unruh T., Weldon G. F., Yu D., Stride J. A., Wood P. T. (2017). Magnetic Properties of the Distorted
Kagomé
Lattice Mn_3_(1,2,4-(O_2_C)_3_C_6_H_3_)_2_. Inorg. Chem..

[48] Pedersen K. S., Perlepe P., Aubrey M. L. (2018). Formation of the Layered
Conductive Magnet CrCl_2_(Pyrazine)_2_ through Redox-Active
Coordination Chemistry. Nat. Chem..

[49] Scarborough C. C., Sproules S., Doonan C. J., Hagen K. S., Weyhermüller T., Wieghardt K. (2012). Scrutinizing
Low-Spin Cr­(II) Complexes. Inorg. Chem..

[50] Rigaku Oxford Diffraction CrysAlis PRO. 2025.

[51] Sheldrick G. M. (2015). SHELXT
– Integrated Space-Group and Crystal-Structure Determination. Acta Crystallogr..

[52] Bourhis L. J., Dolomanov O. V., Gildea R. J., Howard J. a. K., Puschmann H. (2015). The Anatomy
of a Comprehensive Constrained, Restrained Refinement Program for
the Modern Computing Environment – Olex2 Dissected. Acta Crystallogr..

[53] Dolomanov O. V., Bourhis L. J., Gildea R. J., Howard J. A. K., Puschmann H. (2009). OLEX2: A Complete
Structure Solution, Refinement and Analysis Program. J. Appl. Crystallogr..

[54] Saha A., Nia S. S., Rodríguez J. A. (2022). Electron
Diffraction of 3D Molecular
Crystals. Chem. Rev..

[55] Bain G. A., Berry J. F. (2008). Diamagnetic Corrections
and Pascal’s Constants. J. Chem. Educ..

[56] Campbell B. J., Stokes H. T., Tanner D. E., Hatch D. M. (2006). ISODISPLACE: AWeb-Based
Tool for Exploring Structural Distortions. J.
Appl. Crystallogr..

[57] Russina M., Mezei F. (2009). First Implementation of Repetition
Rate Multiplication in Neutron
Spectroscopy. Nucl. Instrum. Methods Phys. Res..

[58] Russina M., Mezei F. (2010). Implementation of Repetition
Rate Multiplication in Cold, Thermal
and Hot Neutron Spectroscopy. J. Phys.: Conf.
Ser..

[59] Arnold O., Bilheux J., Borreguero J. (2014). Mantid-Data Analysis
and Visualization Package for Neutron Scattering and *μ*SR Experiments. Nucl. Instrum. Methods Phys.
Res..

[60] Windsor, C. G. Pulsed Neutron Scattering; Taylor & Francis; Halsted Press: London; New York, 1981.

[61] Clark S. J., Segall M. D., Pickard C. J., Hasnip P. J., Probert M. J., Refson K., Payne M. (2005). First principles
methods using CASTEP. Z. Kristallogr..

[62] Perdew J. P., Burke K., Ernzerhof M. (1996). Generalized
Gradient Approximation
Made Simple. Phys. Rev. Lett..

[63] McNellis E. R., Meyer J., Reuter K. (2009). Azobenzene
at coinage metal surfaces:
Role of dispersive van der Waals interactions. Phys. Rev. B.

[64] Ambrosetti A., Reilly A. M., DiStasio R. A., Tkatchenko A. (2014). Long-range
correlation energy calculated from coupled atomic response functions. J. Chem. Phys..

[65] Monkhorst H. J., Pack J. D. (1976). Special points for
Brillouin-zone integrations. Phys. Rev. B.

[66] Pfrommer B. G., Côté M., Louie S. G., Cohen M. L. (1997). Relaxation of crystals
with the quasi-Newton method. J. Comput. Phys..

[67] Byrd R. H., Nocedal J., Schnabel R. B. (1994). Representations
of quasi-Newton matrices
and their use in limited memory methods. Math.
Prog..

[68] Krukau A.
V., Vydrov O. A., Izmaylov A. F., Scuseria G. E. (2006). Influence of the
exchange screening parameter on the performance of screened hybrid
functionals. J. Chem. Phys..

[69] Morris A. J., Nicholls R. J., Pickard C. J., Yates J. R. (2014). OptaDOS: A tool
for obtaining density of states, core-level and optical spectra from
electronic structure codes. Comput. Phys. Commun..

[70] Nicholls R. J., Morris A. J., Pickard C. J., Yates J. R. (2012). OptaDOS - a new
tool for EELS calculations. J. Phys.: Conf.
Ser..

[71] Yates J. R., Wang X., Vanderbilt D., Souza I. (2007). Spectral and Fermi
surface properties from Wannier interpolation. Phys. Rev. B.

[72] Evans M., Morris A. (2020). matador: a Python library for analysing, curating and
performing high-throughput density-functional theory calculations. J. Open Source Software.

[73] Rutter M. (2018). C2x: A tool
for visualisation and input preparation for Castep and other electronic
structure codes. Comput. Phys. Commun..

[74] Fonari, A. ; Sutton, C. Effective Mass Calculator for Semiconductors 2012 https://github.com/afonari/emc (accessed April 24, 2026).

