# Supplementary material for: Enhancing Superexchange through Frontier Orbital Engineering in a van der Waals Metal–Organic Magnet
Source: Chem Mater. 2026 May 26;38(11):5649–57. doi: 10.1021/acs.chemmater.6c00516 (PMC13255171; doi:10.1021/acs.chemmater.6c00516)
Supplement: Supplementary file 1 [file cm6c00516_si_001.pdf]

Enhancing superexchange through frontier orbital engineering in a van der Waals metal-organic magnet

Jem Pitcairn[a], Mario Antonio T. Ongkiko[b], Peter J. Speakman[a], Jeremiah P. Tidey[c], Jack Jordan[d], Graham N. Newton[c], Pascal Manuel[e], J. Ross Stewart[e], Andrew J. Morris[b], and Matthew J. Cliffe [*a,f]

[a]School of Chemistry, University of Nottingham, University Park, Nottingham, NG7 2RD, United Kingdom
[b]School of Metallurgy and Materials, University of Birmingham, Elms Rd, Edgbaston, Birmingham B15 2TT, United Kingdom
[c]Department of Physics, University of Warwick, Gibbet Hill Road, Coventry, CV4 7AL, United Kingdom
[d]Carbon Neutral Laboratory, School of Chemistry, University of Nottingham, Jubilee Campus, Nottingham, NG7 2GT, United Kingdom
[e]ISIS Neutron and Muon Source, STFC Rutherford Appleton Laboratory, Didcot OX11 0QX, United Kingdom
[f]Department of Materials Science and Metallurgy, University of Cambridge, 27 Charles Babbage Road, Cambridge CB3 OFS, United Kingdom

April 27, 2026

Contents

List of Figures 1

List of Tables 2

S1 Powder diffraction 4

S2 Electron crystallography 5

S3 Density-functional theory 8

S4 Magnetism 14

S5 Cyclic voltammetry 18

List of Figures

S1 Rietveld refinement against PXRD data of $CrCl_2$(btd). 4
S2 Magnetic structure of $CrCl_2$(btd$-$d$_4$) viewed along the [001] direction 4
S3 Magnetic structure of $CrCl_2$(btd$-$d$_4$) viewed along the [010] direction 5

*mjc222@cam.ac.uk

S4 Diffraction plane reconstructions for the $0kl$ (top), $hk\bar{1}$ (middle), and $hk-2.5$ (bottom) planes for the major (left) and doubled-cell minor phase (right), all presented compared to the indexed to the major monoclinic-*P* unit cell of with *a* = 3.73 Å, *b* = 12.87 Å, *c* = 8.82 Å and *β* = 96.1° with identical processing and visualisation parameters. Note in particular the difference in periodicity in the vertical direction for the $0kl$ images (doubled for the minor phase), and the distinct absence of reflections in the $hk-2.5$ image for the major phase, indicative of the doubling of the *c* axis in the minor phase. Weak contaminating intensities due to significant mosaicity and minor crystallites in the area selection are present. 7
S5 Spin density isosurfaces (0.01 e Å^{-3}) of the $CrCl_2$(btd) lower energy magnetic supercell derived from CASTEP PBE+U+MBD* (U_{eff} = 3 eV) and C2X calculations. The colour scheme differentiates between positive (orange) and negative (green) spin densities. . 8
S6 Spin density isosurfaces (0.01 e Å^{-3}) of the $CrCl_2$(pym) lower energy magnetic supercell, derived from CASTEP PBE+U+MBD* (U_{eff} = 3 eV) and C2X calculations. The colour scheme differentiates between positive (orange) and negative (green) spin densities. . 8
S7 Visualisation of the HOMO of $CrCl_2$(btd). The 'up' spin channel was calculated at the Γ point using the DFT package CASTEP along with C2X. Blue and yellow correspond to the positive and negative phases of the Kohn-Sham orbitals. 9
S8 Visualisation of the LUMO of $CrCl_2$(btd). The 'up' spin channel was calculated at the Γ point using the DFT package CASTEP along with C2X. Blue and yellow correspond to the positive and negative phases of the Kohn-Sham orbitals. 9
S9 Visualisation of the HOMO of $CrCl_2$(pym). The 'up' spin channel was calculated at the Γ point using the DFT package CASTEP along with C2X. Blue and yellow correspond to the positive and negative phases of the Kohn-Sham orbitals. 10
S10 Visualisation of the LUMO of $CrCl_2$(pym). The 'up' spin channel was calculated at the Γ point using the DFT package CASTEP along with C2X. Blue and yellow correspond to the positive and negative phases of the Kohn-Sham orbitals. 10
S11 The band structure and projected density of states of $CrCl_2$(btd) using the lower energy magnetic supercell using CASTEP 23.1 at the PBE+U+MBD* (U_{eff}= 3 eV) level. Density of states has been projected over LCAO states using OPTADOS. 10
S12 The projected density of states of $CrCl_2$(btd) using the structural cell using CASTEP 23.1 at the HSE06 level. Density of states has been projected over LCAO states using OPTADOS. 11
S13 The band structure and projected density of states of $CrCl_2$(pym) using the lower energy magnetic supercell using CASTEP 23.1 at the PBE+U+MBD* (U_{eff}= 3 eV) level. Density of states has been projected over LCAO states using OPTADOS. 11
S14 The projected density of states of $CrCl_2$(pym) using the structural cell using CASTEP 23.1 at the HSE06 level. Density of states has been projected over LCAO states using OPTADOS. 11
S15 Fitting of the isothermal magnetisation of $CrCl_2$btd$-$d$_4$ measured at 2K with a Brillouin function and linear antiferromagnetic component 14
S16 INS spectra and linear spin wave fit global and local minima for $CrCl_2$(btd). 15
S17 INS spin wave fit metric for $CrCl_2$(btd). 16
S18 Further INS spectra of $CrCl_2$(btd). 17
S19 Cyclic voltammagrams of btd carried out in 1M LiTFSI dissolved in MeCN with an analyte concentration of 1 mM 18
S20 Cyclic voltammagrams of pym carried out in 1M LiTFSI dissolved in MeCN with an analyte concentration of 1 mM 19
S21 Comparison of the cyclic voltammagrams for pym and btd carried out in 1M LiTFSI dissolved in MeCN with an analyte concentration of 1 mM 20

List of Tables

S1 Summary of electron crystallography results. 5
S2 Calculated lattice parameters from PBE+U+MBD* DFT. 12
S3 Calculated magnetic superexchange from PBE+U+MBD*. 12
S4 Calculated metal-ligand band gap, E_g, and bandwidth W of the organic ligand LUMO for different functionals. 12
S5 Calculated effective masses (in m_e) of the organic ligand conduction band ($m_{X,C}$) and Cr-Cl valence band ($m_{X,V}$) from the lowest energy magnetic supercell calculated using CASTEP PBE+U+MBD* (U_{eff} = 3 eV) at Γ (0, 0, 0) with 0.01 Bohr step size for differentiation. m_1 lies approximately along the a^* direction, corresponding to the $\pi - \pi$ stacking of the ligands, m_2 lies approximately along the b^* direction, corresponding to the alternating up and down ligands, and m_3 lies approximately along the c^* direction, corresponding to the interlayer stacking. 13
S6 Magnetic property parameters from susceptibility. 14

S1 Powder diffraction

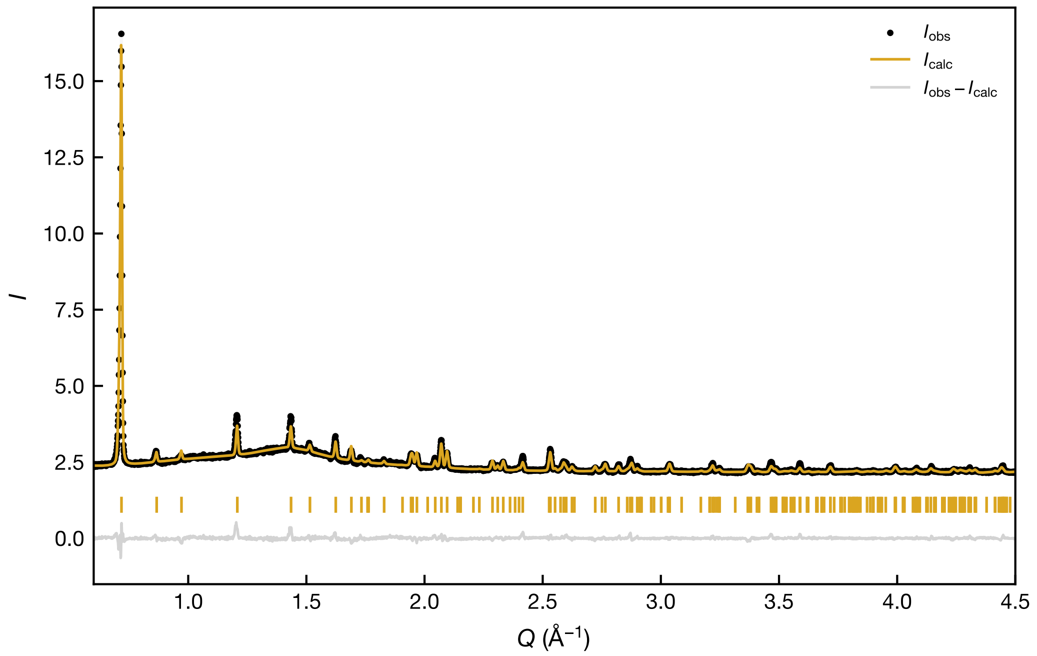

Figure S1: Rietveld refinement[1] of powder X-ray diffraction data of $CrCl_2$(btd) collected in ambient conditions on a PANalytical X'Pert Pro diffractometer equipped with monochromated Cu Kα_1 radiation ($\lambda = 1.5406$ Å).

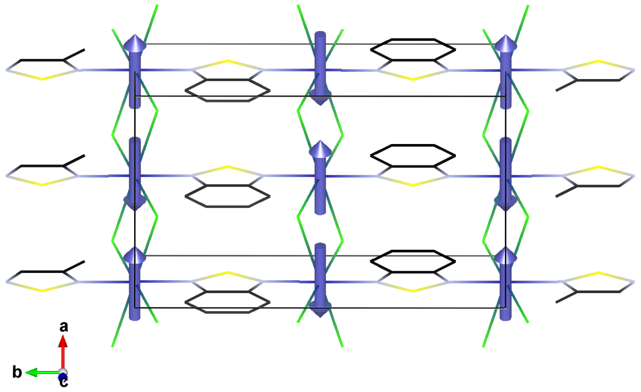

Figure S2: Magnetic structure of $CrCl_2$(btd$-d_4$) viewed along the [001] direction

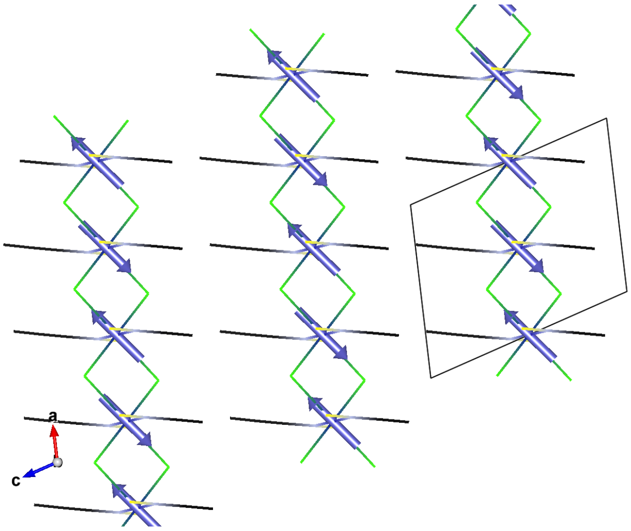

Figure S3: Magnetic structure of $CrCl_2$(btd$-d_4$) viewed along the [010] direction

S2 Electron crystallography

Two phases were identified in the sample, the dominant phase discussed in the main text, $CrCl_2$(btd), and a minor polymorph with a double *c* axis, here $CrCl_2$(btd)-2*c*, of which only two crystals were identified. In the case of $CrCl_2$(btd)-2*c*, a twin law was required to describe the pseudo-merohedral twinning of the $2/m$ phase which forms with a lattice having mmm symmetry (C-centred). Consequently, component datasets were first individually integrated and solved to ascertain their twin batch scale factors and two crystals having similar twin compositions were merged, resulting in a dataset that is self-consistent in its twin-law description. This approach allowed for both the twinning to be explicitly handled and for merging to average the impact of multiple scattering to improve its description by use of the extinction correction, resulting in a significant improvement against the use of individual datasets. Reconstructed diffraction planes clearly show the presence of these additional reflections (Fig. S4)

In both cases, all non-H atoms are refined anisotropically, while hydrogen atoms were placed geometrically with bond distances refined in the presence of distance similarity restraints and isotropic displacement parameters. In the case of $CrCl_2$(btd), no further restraints or constraints are applied. For $CrCl_2$(btd)-2*c*, a global rigid bond restraint and distance similarity restraints across chemically equivalent bonds were applied to improve the robustness of the model to the combination of twinning and multiple diffraction, alongside additional displacement similarity restraints on the C-H pairs in one of the asymmetric molecules. Experimental and refinement information are contained within the deposited CIF along with structure factors and embedded .RES files; structure CIFs are deposited in the CSD with Deposition Numbers 2457667-2457668.

Table S1: Summary of electron crystallography results.

Crystal Data	$CrCl_2$(btd)	$CrCl_2$(btd)-2c
Chemical formula	$C_6H_4Cl_2CrN_2S$	$C_6H_4Cl_2CrN_2S$
M_r (Da)	259.08	259.08
Crystal system	Monoclinic	Monoclinic
Space group	$P2_1/m$	$P2_1/m$
Temperature (K)	293(2)	293(2)
a (Å)	3.7180(6)	3.7297(3)
b (Å)	12.9099(13)	12.9171(9)
c (Å)	8.7866(19)	17.586(3)
β (°)	96.149(17)	96.146(10)

Crystal Data	$CrCl_2$(btd)	$CrCl_2$(btd)-2c
V (Å^3)	419.32 (12)	842.36 (16)
Z'	0.5	1
Radiation type	Electron	Electron
λ (Å)	0.02510	0.02510
Scan range (°)	See CIF	See CIF
Measured reflections	14455	9126
Independent reflections	1643	1974
Observed ($I \geq 2u(I)$) reflections	1236	1500
R_{int}	0.26	0.151
sin$\theta_{\text{max}}/\lambda$ (Å^{-1})	0.786	0.695
R_1 ($F^2 > 2\sigma(F^2)$)	0.1629	0.1381
wR_2 ($F^2 > 2\sigma(F^2)$)	0.3631	0.3198
R_1 (all)	0.1956	0.1606
wR_2 (all)	0.3827	0.3357
GoF (S) (inc. restraints)	1.0287	1.0119
GoF (S) (excl. restraints)	1.0287	1.0404
No. reflections	1643	1974
No. parameters	63	125
No. restraints	2	106
$\Delta\phi_{\text{max}}$ (as reported by Olex2.refine, AC07-014)	1.14	1.06
$\Delta\phi_{\text{min}}$ (as reported by Olex2.refine, AC07-014)	−1.32	−0.83

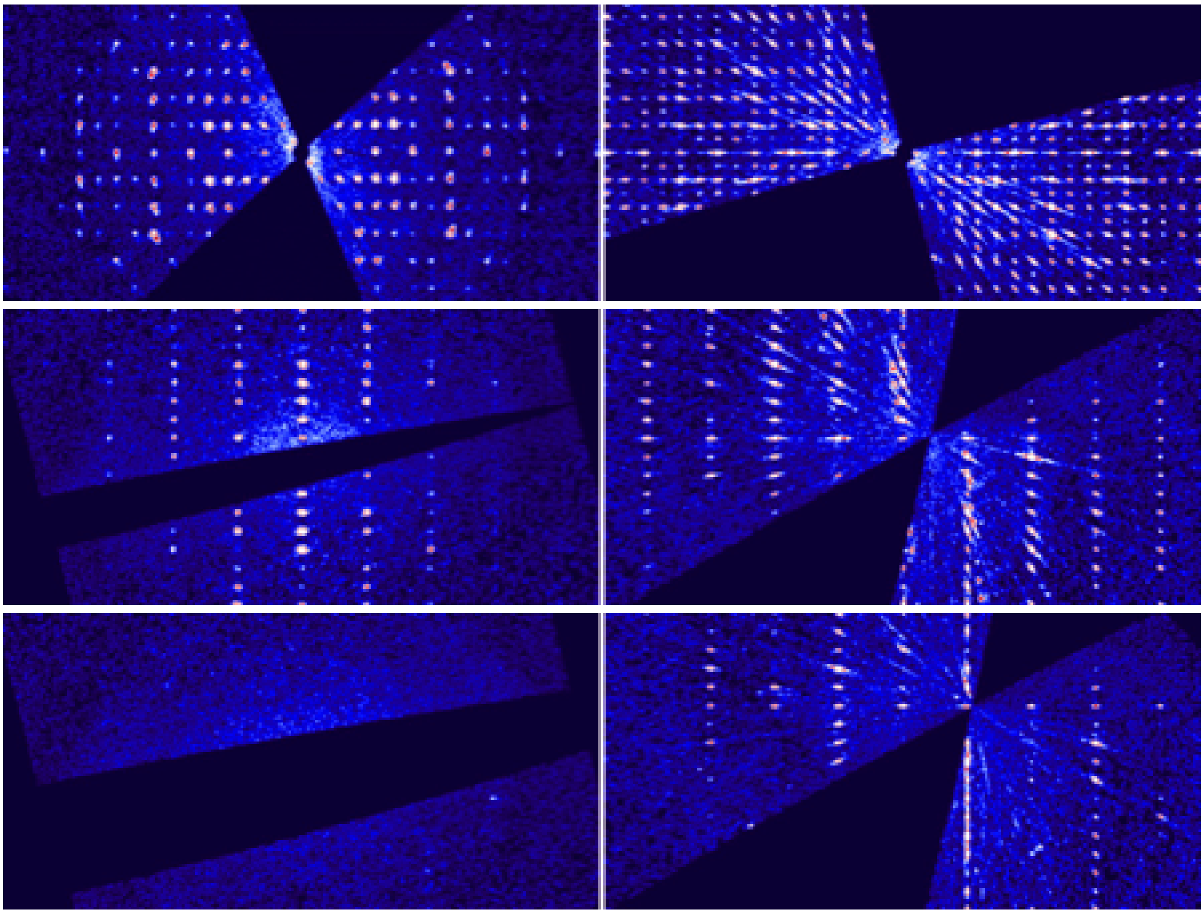

Figure S4: Diffraction plane reconstructions for the $0kl$ (top), $hk\bar{1}$ (middle), and $hk-2.5$ (bottom) planes for the major (left) and doubled-cell minor phase (right), all presented compared to the indexed to the major monoclinic-*P* unit cell of with *a* = 3.73 Å, *b* = 12.87 Å, *c* = 8.82 Å and *β* = 96.1° with identical processing and visualisation parameters. Note in particular the difference in periodicity in the vertical direction for the $0kl$ images (doubled for the minor phase), and the distinct absence of reflections in the $hk-2.5$ image for the major phase, indicative of the doubling of the *c* axis in the minor phase. Weak contaminating intensities due to significant mosaicity and minor crystallites in the area selection are present.

S3 Density-functional theory

The structural relaxation of $CrCl_2$btd revealed the choice of Hubbard U determined whether Jahn-Teller (JT) distortion manifested in octahedral Cr^{2+}. An initial structure for $CrCl_2$(btd) was constructed by swapping Ni in $NiCl_2$(btd) with Cr. This non-JT structure was then relaxed with a $U_{\text{eff}} = 0$ eV; however, the resulting structure did not have any JT distortion. The structure was then relaxed for a range of $U_{\text{eff}} = 1, 3, 5, 7$,eV. Among these, $U_{\text{eff}} = 5, 7$ eV relaxations led to JT-distorted structures. We tested the robustness of the JT-distortion with respect to an empirically chosen U_{eff}. The JT-distorted U_{eff},$= 5$ eV structure was used to aid in Rietveld refinement of the experimental structure. This experimentally refined structure was further relaxed with $U_{\text{eff}} = 0, 1, 3, 7$ eV. Table S2 shows the calculated lattice parameters for the $U_{\text{eff}} = 3$ eV JT-distorted structures for both $CrCl_2$(btd) and $CrCl_2$(pym). The JT distortion was maintained for $U_{\text{eff}} = 3\&7$ eV relaxations, while the distortion was absent for $U_{\text{eff}} = 0\&1$ eV. This suggested $U_{\text{eff}} = 3$ eV was an approximate transition point for the JT distortion in DFT+U calculations of $CrCl_2$(btd).

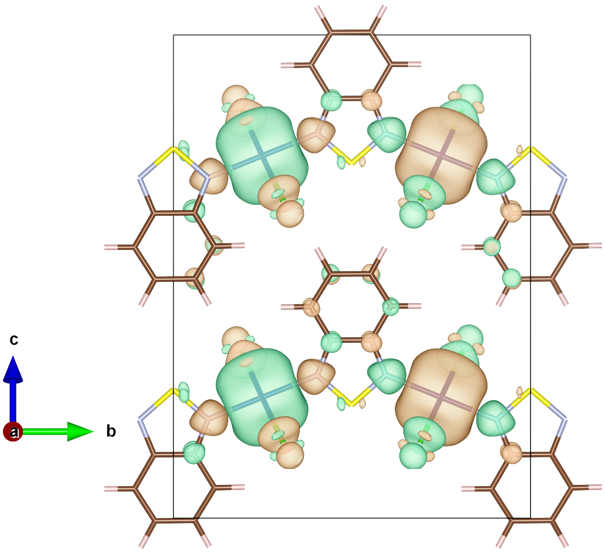

Figure S5: Spin density isosurfaces (0.01 e Å^{-3}) of the $CrCl_2$(btd) lower energy magnetic supercell derived from CASTEP PBE+U+MBD* (U_{eff} = 3 eV) and C2X calculations. The colour scheme differentiates between positive (orange) and negative (green) spin densities.

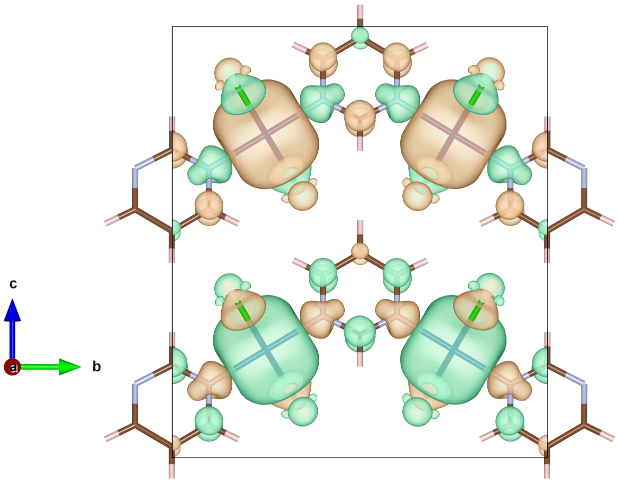

Figure S6: Spin density isosurfaces (0.01 e Å^{-3}) of the $CrCl_2$(pym) lower energy magnetic supercell, derived from CASTEP PBE+U+MBD* (U_{eff} = 3 eV) and C2X calculations. The colour scheme differentiates between positive (orange) and negative (green) spin densities.

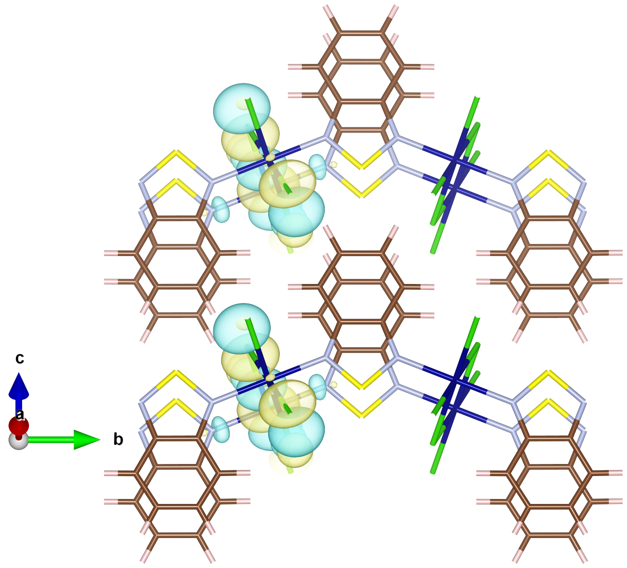

Figure S7: Visualisation of the HOMO of $CrCl_2$(btd). The 'up' spin channel was calculated at the Γ point using the DFT package CASTEP along with C2X. Blue and yellow correspond to the positive and negative phases of the Kohn-Sham orbitals.

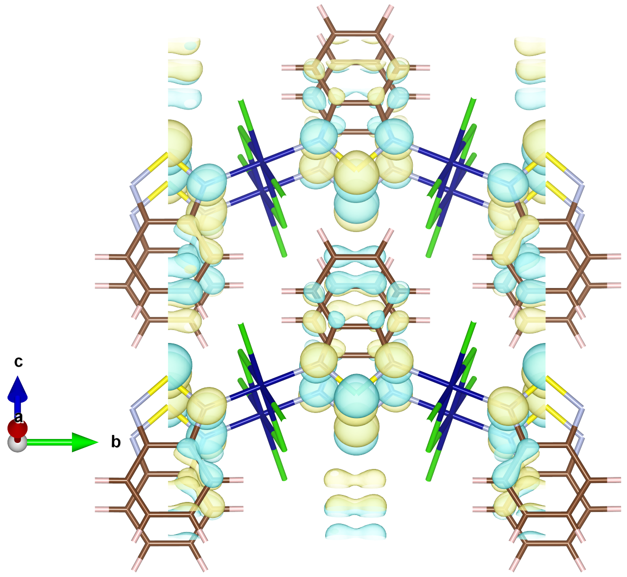

Figure S8: Visualisation of the LUMO of $CrCl_2$(btd). The 'up' spin channel was calculated at the Γ point using the DFT package CASTEP along with C2X. Blue and yellow correspond to the positive and negative phases of the Kohn-Sham orbitals.

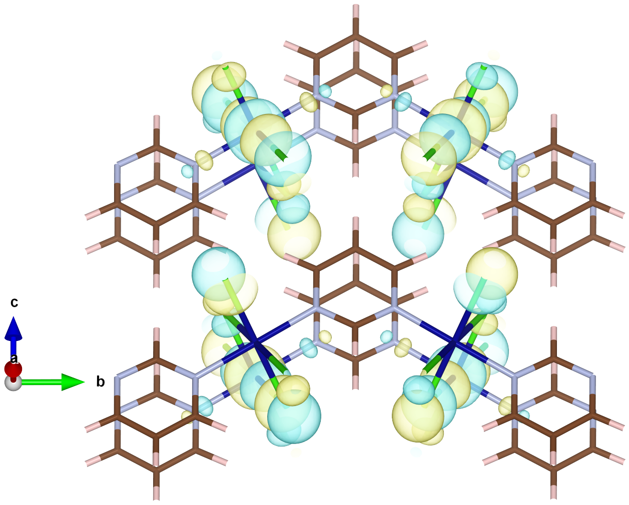

Figure S9: Visualisation of the HOMO of $CrCl_2$(pym). The 'up' spin channel was calculated at the Γ point using the DFT package CASTEP along with C2X. Blue and yellow correspond to the positive and negative phases of the Kohn-Sham orbitals.

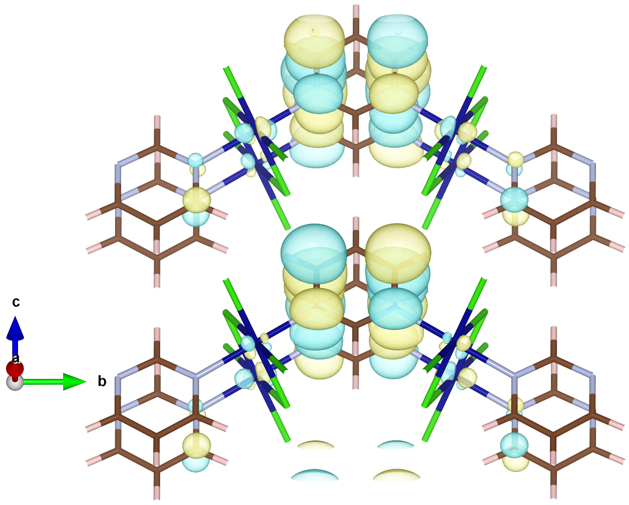

Figure S10: Visualisation of the LUMO of $CrCl_2$(pym). The 'up' spin channel was calculated at the Γ point using the DFT package CASTEP along with C2X. Blue and yellow correspond to the positive and negative phases of the Kohn-Sham orbitals.

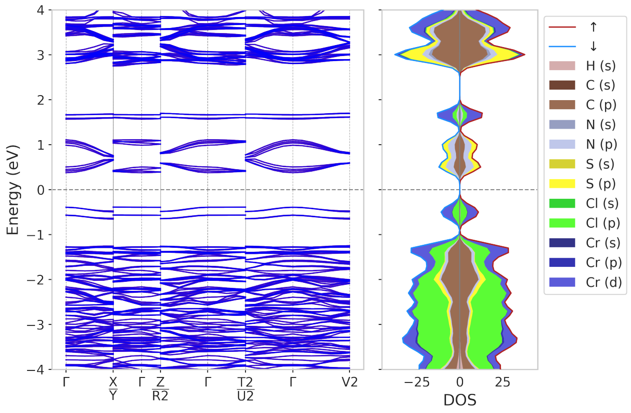

Figure S11: The band structure and projected density of states of $CrCl_2$(btd) using the lower energy magnetic supercell using CASTEP 23.1 at the PBE+U+MBD* (U_{eff}= 3 eV) level. Density of states has been projected over LCAO states using OPTADOS.

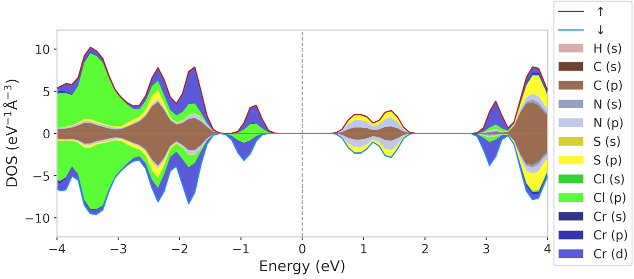

Figure S12: The projected density of states of $CrCl_2$(btd) using the structural cell using CASTEP 23.1 at the HSE06 level. Density of states has been projected over LCAO states using OPTADOS.

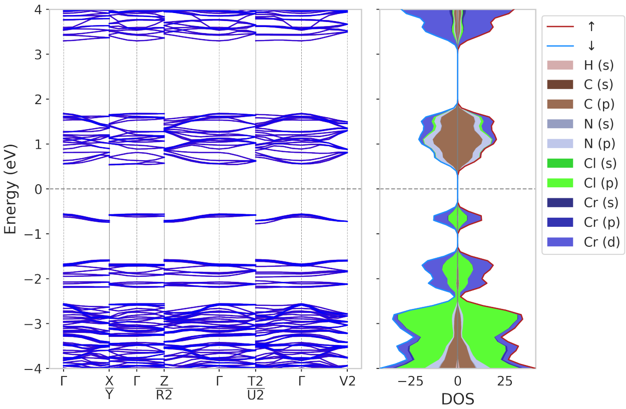

Figure S13: The band structure and projected density of states of $CrCl_2$(pym) using the lower energy magnetic supercell using CASTEP 23.1 at the PBE+U+MBD* (U_{eff}= 3 eV) level. Density of states has been projected over LCAO states using OPTADOS.

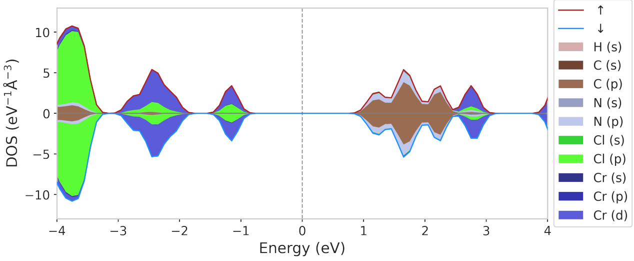

Figure S14: The projected density of states of $CrCl_2$(pym) using the structural cell using CASTEP 23.1 at the HSE06 level. Density of states has been projected over LCAO states using OPTADOS.

Table S2: Calculated lattice parameters from PBE+U+MBD* DFT.

	$CrCl_2$(btd)				$CrCl_2$(pym)			
U (eV)	1	3	5	7	1	3	5	7
a (Å)	3.60	3.79	3.82	3.83	3.65	3.72	3.79	3.83
b (Å)	12.67	12.81	12.89	12.94	11.99	12.07	12.13	12.20
c (Å)	9.29	9.19	9.22	9.25	6.98	6.99	6.92	6.92
β (°)	111.37	109.43	109.60	109.83	95.32	96.56	97.27	97.57
V (Å^3)	394.71	421.05	426.97	431.60	303.96	312.20	315.63	320.87
Bond lengths (Å)								
Cr-Cl1	2.37	2.37	2.40	2.43	2.39	2.42	2.44	2.48
Cr-Cl2	2.37	2.94	2.98	2.98	2.74	2.84	2.88	2.90
Cr-N	2.09	2.14	2.17	2.18	2.12	2.14	2.17	2.19

Table S3: Calculated magnetic superexchange from PBE+U+MBD*.

	$CrCl_2$(btd)			
U(eV)	1	3	5	7
J_{Cl} (meV)	-1.4(2)	-1.36(3)	-0.73(1)	-0.30(1)
J_{btd} (meV)	-2.3(2)	-1.59(3)	-1.03(1)	-0.75(1)
J_{vdW} (meV)	0.0(2)	-0.02(3)	-0.01(1)	-0.00(1)
$\|J_{Cl}/J_{btd}\|$	0.58	0.86	0.72	0.41

	$CrCl_2$(pym)			
U (eV)	1	3	5	7
J_{Cl} (meV)	-1.87(7)	-1.17(3)	-0.70(1)	-0.29(1)
J_{pym} (meV)	0.41(7)	0.23(3)	0.13(1)	0.06(1)
J_{vdW} (meV)	-0.11(7)	-0.04(3)	-0.02(1)	-0.01(1)
$\|J_{Cl}/J_{pym}\|$	4.59	5.07	5.23	4.59

Table S4: Calculated metal-ligand band gap, E_g, and bandwidth W of the organic ligand LUMO for different functionals.

	$CrCl_2$(btd)				
U (eV)	1	3	5	7	HSE06
E_g (eV)	0.42	0.77	1.11	1.35	1.51
W (eV)	0.8	0.8	0.8	0.8	–

	$CrCl_2$(pym)				
U (eV)	1	3	5	7	HSE06
E_g	0.62	1.10	1.48	1.74	2.19
W	1.1	1.2	1.2	1.1	–

Table S5: Calculated effective masses (in m_e) of the organic ligand conduction band ($m_{X,C}$) and Cr-Cl valence band ($m_{X,V}$) from the lowest energy magnetic supercell calculated using CASTEP PBE+U+MBD* (U_{eff} = 3 eV) at Γ (0, 0, 0) with 0.01 Bohr step size for differentiation. m_1 lies approximately along the a^* direction, corresponding to the $\pi - \pi$ stacking of the ligands, m_2 lies approximately along the b^* direction, corresponding to the alternating up and down ligands, and m_3 lies approximately along the c^* direction, corresponding to the interlayer stacking.

	$CrCl_2$(btd)			
U (eV)	1	3	5	7
$m_{1,C}$	1.884	1.907	1.918	1.925
$m_{2,C}$	50.929	6.882	5.171	4.401
$m_{3,C}$	7.179	7.597	8.214	8.310
$m_{1,V}$	-4.109	-2.463	-1.777	-1.233
$m_{2,V}$	40.429	37.024	33.473	56.617
$m_{3,V}$	-40.026	-43.981	-47.201	-29.571

	$CrCl_2$(pym)			
U (eV)	1	3	5	7
$m_{1,C}$	3.166	4.002	2.357	2.242
$m_{2,C}$	-19.186	-4.254	-3.656	-3.708
$m_{3,C}$	-1.074	-0.376	-0.930	-1.991
$m_{1,V}$	-2.765	-1.830	-1.372	-1.134
$m_{2,V}$	-12.325	-8.869	-7.642	-7.300
$m_{3,V}$	-34.419	-36.267	-36.109	-37.375

S4 Magnetism

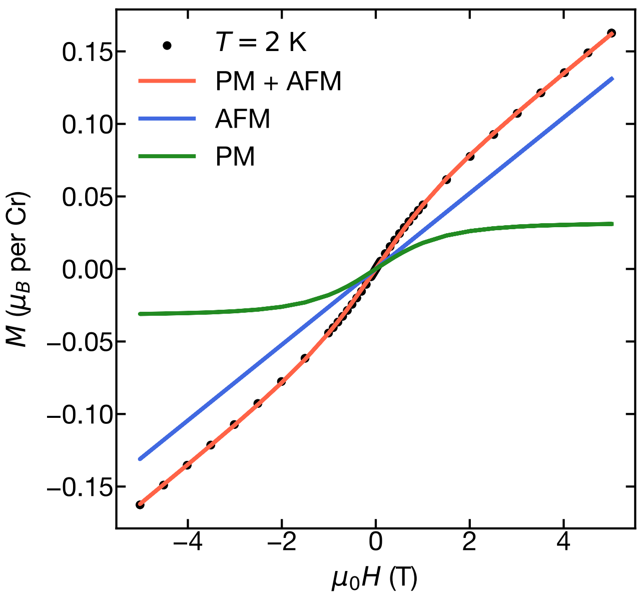

Figure S15: Fitting of the isothermal magnetisation of $CrCl_2btd{-}d_4$ measured at 2K with a Brillouin function and linear antiferromagnetic component

Table S6: Magnetic property parameters determined from magnetic susceptibility measurements with the 1D Heisenberg AFM Fisher,[2] 2D quadratic Heisenberg AFM Lines'[3] and Curie-Weiss mean-field model. The Fisher and Lines' models were fit to data at $48 < T < 300$ K and the Curie-Weiss model was fit to data at $200 < T < 300$ K.

	Fisher	Lines'	Curie-Weiss
g	2.33(1)	2.15(10)	2.16(6)
J (K)	-139(2)	-9.6(5)	–
θ_{CW} (K)	-554(6)	-77.2(2)	-86(2)

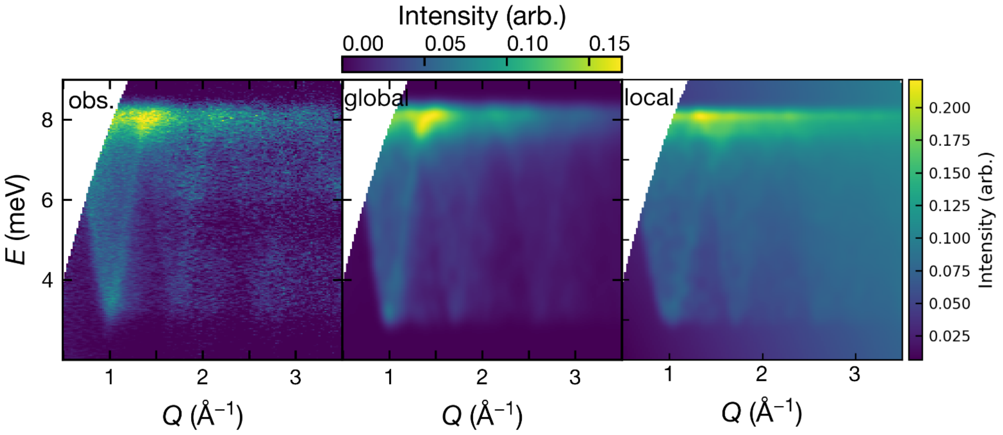

Figure S16: Left: Time-of-flight powder INS spectra of $CrCl_2(btd{-}d_4)$ with $E_i = 12.14$ meV measured at 1.7 K.[4]. Centre: LSWT calculated scattering intensity fitted to the 1.7 K data, with parameters $J_{\text{Cl}} = -15.31(84)$, $J_{\text{btd}} = -6.96(54)$, $J_{\text{vdW}} = -0.1(1)$ and $D = -2.0(2)$ K. Right: LSWT calculated scattering intensity fitted to the 1.7 K data, with parameters $J_{\text{Cl}} = -6.96$, $J_{\text{btd}} = -15.31$, $J_{\text{vdW}} = -0.1$ and $D = -2.0$ K. This fit corresponds to the local minimum with J_{Cl} and J_{btd} swapped compared to the global minimum. Hamiltonian given by $\mathcal{H} = \sum_{\langle ij \rangle} -J_{ij}\mathbf{S}_i \cdot \mathbf{S}_j + \sum_i D(S_i^z)^2$.

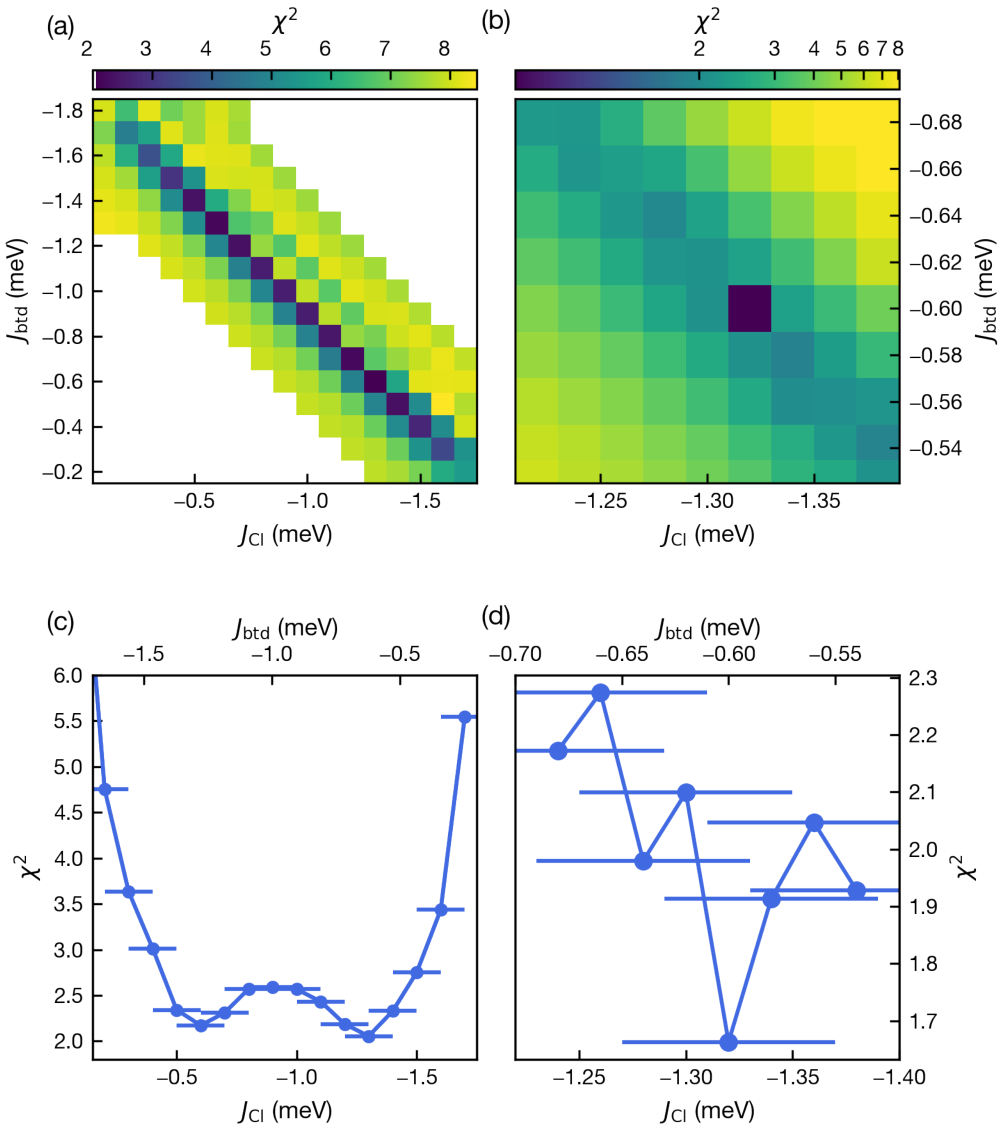

Figure S17: Reduced χ^2 as a function of J_{Cl} and J_{btd} where $J_{\text{vdW}} = -0.01(1)$ meV and $D = -0.17(3)$ meV over (a) $-1.8 < J < -0.2$ meV in 0.1 meV increments and (b) $-1.38 < J_{\text{Cl}} < -1.22$ meV and $- < J_{\text{btd}} < -0.52$. The reduced χ^2 values for (c) $J_{\text{Cl}} + J_{\text{btd}} = -1.9$ meV in the 0.1 meV resolution grid search and (d) $J_{\text{Cl}} + J_{\text{btd}} = -1.92$ meV in the 0.02 meV resolution grid search. Error bars in (c) are the resolution of the array search and the error bars in (d) are the combined error for J_{Cl} and J_{btd}.

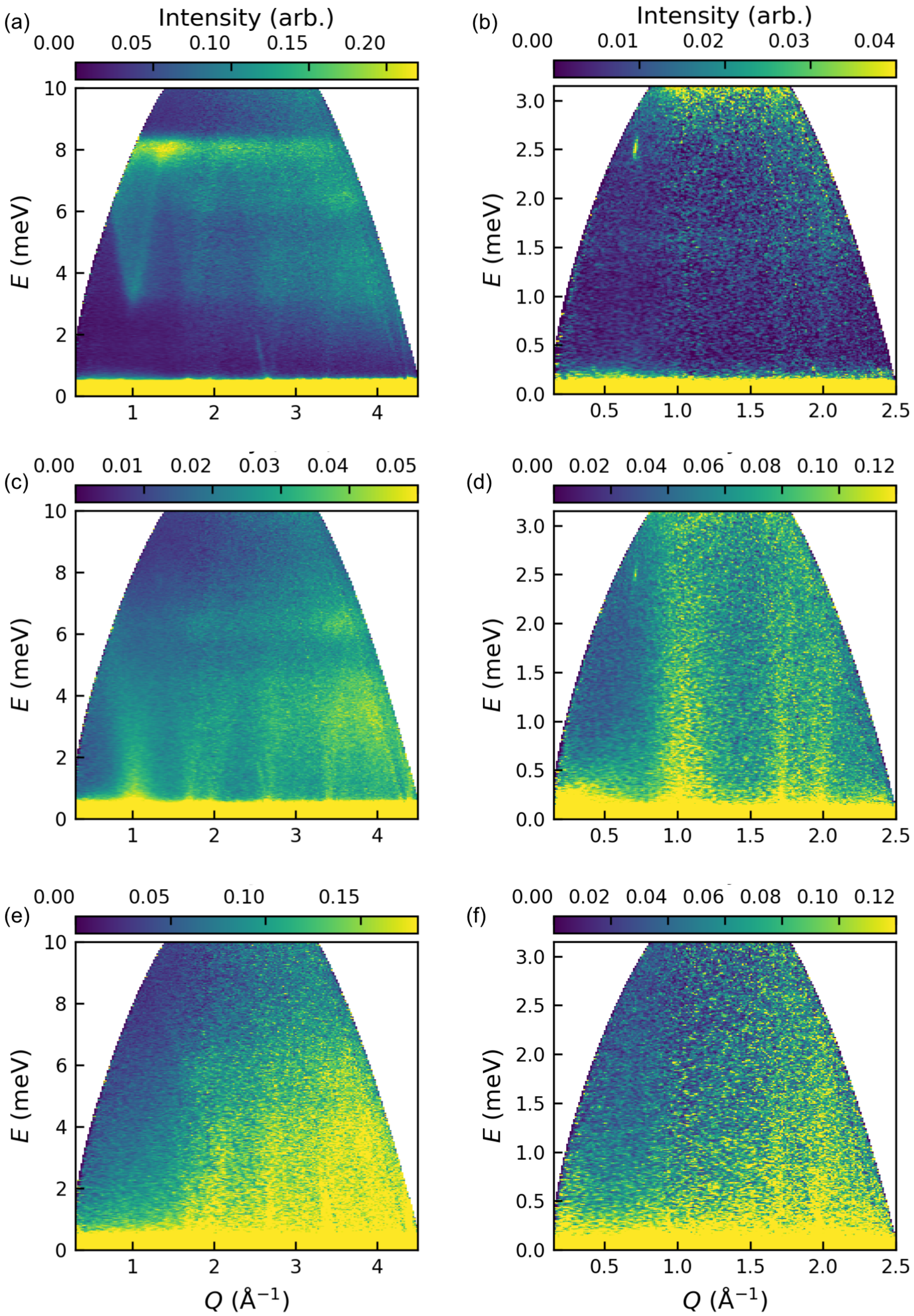

Figure S18: Image plot of INS intensity as a function of energy and wave-vector transfer for $CrCl_2(btd{-}d_4)$ at: (a) $E_i = 12.14$ meV, $T = 1.7$ K; (b) $E_i = 3.71$ meV, $T = 1.7$ K; (c) $E_i = 12.14$ meV, $T = 60$ K; (d) $E_i = 3.71$ meV, $T = 60$ K; (e) $E_i = 12.14$ meV, $T = 285$ K; (f) $E_i = 3.71$ meV, $T = 285$ K.[4]

S5 Cyclic voltammetry

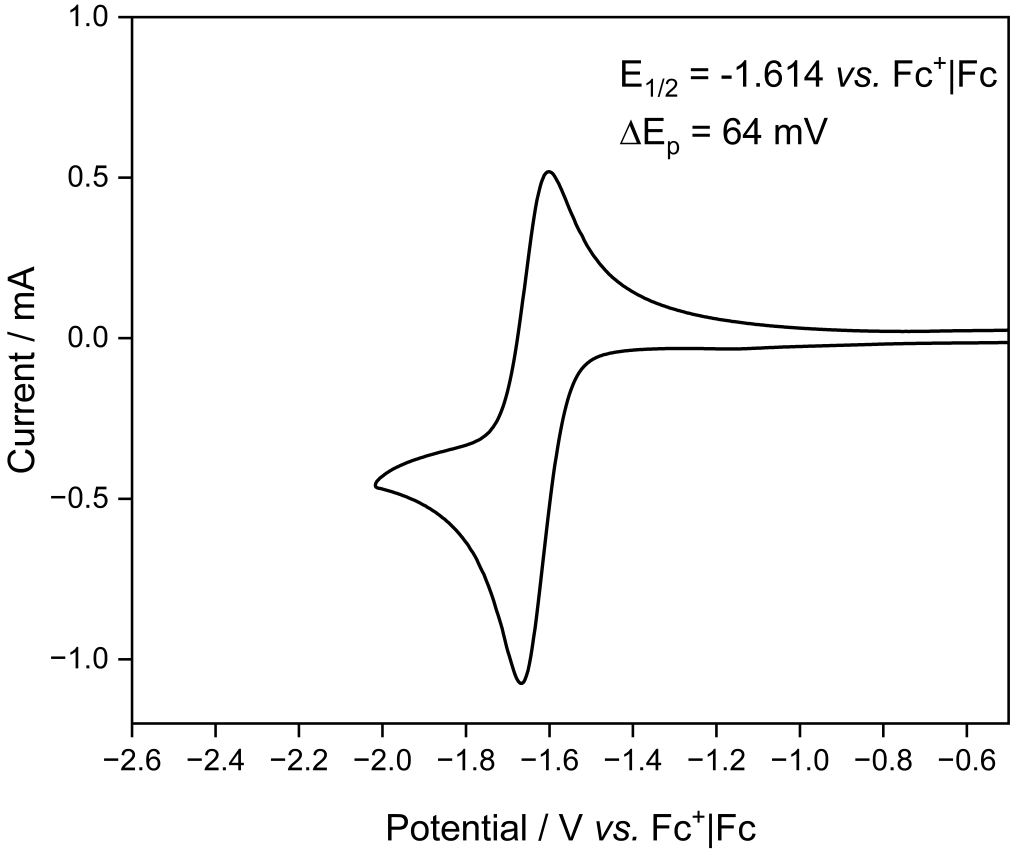

Figure S19: Cyclic voltammagrams of btd carried out in 1M LiTFSI dissolved in MeCN with an analyte concentration of 1 mM

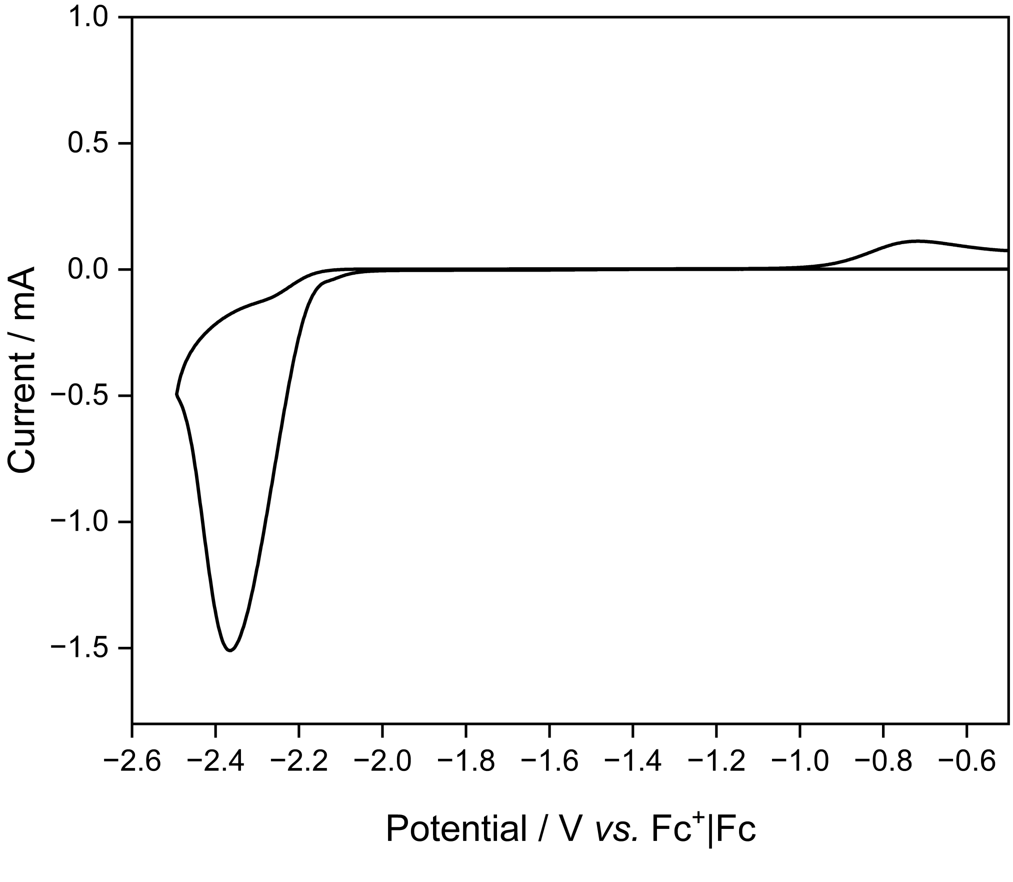

Figure S20: Cyclic voltammagrams of pym carried out in 1M LiTFSI dissolved in MeCN with an analyte concentration of 1 mM

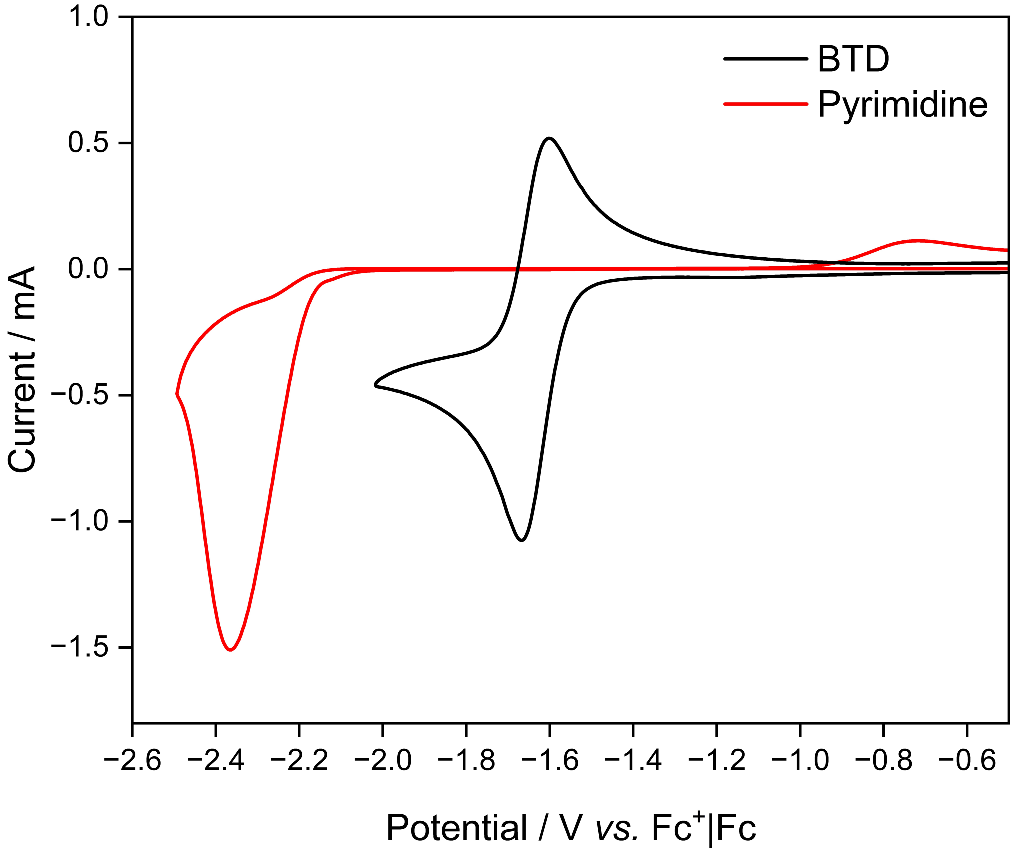

Figure S21: Comparison of the cyclic voltammagrams for pym and btd carried out in 1M LiTFSI dissolved in MeCN with an analyte concentration of 1 mM

References

[1] Rietveld, H. M. A Profile Refinement Method for Nuclear and Magnetic Structures. *J. Appl. Cryst.* **1969**, *2*, 65–71.

[2] Fisher, M. E. Magnetism in One-Dimensional Systems-The Heisenberg Model for Infinite Spin. *Am. J. Phys.* **1964**, *32*, 343–346.

[3] Lines, M. E. The Quadratic-Layer Antiferromagnet. *J. Phys. Chem. Solids* **1970**, *31*, 101–116.

[4] Chapon, L. C.; Manuel, P.; Radaelli, P. G.; Benson, C.; Perrott, L.; Ansell, S.; Rhodes, N. J.; Raspino, D.; Duxbury, D.; Spill, E.; Norris, J. Wish: The New Powder and Single Crystal Magnetic Diffractometer on the Second Target Station. *Neutron News* **2011**, *22*, 22–25.
