# Supplementary material for: Enhancing Superexchange through Frontier Orbital Engineering in a van der Waals Metal–Organic Magnet
Source: Chem Mater. 2026 May 26;38(11):5649–57. doi: 10.1021/acs.chemmater.6c00516 (PMC13255171; doi:10.1021/acs.chemmater.6c00516)

checkCIF/PLATON report

Structure factors have been supplied for datablock(s) m2_516_517_L072_R07

THIS REPORT IS FOR GUIDANCE ONLY. IF USED AS PART OF A REVIEW PROCEDURE FOR PUBLICATION, IT SHOULD NOT REPLACE THE EXPERTISE OF AN EXPERIENCED CRYSTALLOGRAPHIC REFEREE.

No syntax errors found. CIF dictionary Interpreting this report

Datablock: m2_516_517_L072_R07

```
Bond precision:    C-C = 0.0106 A           Wavelength=0.02510

Cell:              a=3.7297(3)     b=12.9171(9)        c=17.586(3)
                   alpha=90        beta=96.146(10)     gamma=90
Temperature:       293 K
```

	Calculated	Reported
Volume	842.37(17)	842.36(16)
Space group	P 21/m	P 1 21/m 1
Hall group	-P 2yb	-P 2yb
Moiety formula	C6 H4 Cl2 Cr N2 S	2(C3 H2 Cl Cr0.5 N S0.5)
Sum formula	C6 H4 Cl2 Cr N2 S	C6 H4 Cl2 Cr N2 S
Mr	259.07	259.08
Dx,g cm-3	2.043	2.043
Z	4	4
Mu (mm-1)	0.000	0.000
F000	0.0	173.7
F000'	511.92	
h,k,lmax	5,17,24	5,17,21
Nref	2472	1974
Tmin,Tmax		
Tmin'		

Correction method= Not given

Data completeness= 0.799 Theta(max)= 1.000

R(reflections)= 0.1381(1500) wR2(reflections)= 0.3357(1974)

S = 1.040 Npar= 125

The following ALERTS were generated. Each ALERT has the format
test-name_ALERT_alert-type_alert-level.
Click on the hyperlinks for more details of the test.

Alert level B

PLAT110_ALERT_2_B ADDSYM Detects Potential Lattice Translation ... ? Check

Author Response: This sample is a small distortion superstructure of a parent phase with c/2 phase. The closeis relationship to the higher symmetry relative phase is what is detected by ADDSYMM. The reflections due to the doubling of the c axis can be clearly seen in the raw data and are not seen in the datasets of the c/2 phase. We are, therefore, able to confidently assign the supercell. We note that there is no mechanism by which these intensities can arise from multiple scattering. Similarly, while the observed twinning would produce reflections with l odd (i.e. reflections indicative of cell doublinge) for h odd, we also observe reflections with l odd for h even. Thus, twinning of the primary phase cannot account for the observed diffraction. We note finally that, although the diffraction falls on a centred lattice of orthorhombic metric symmetry, it was not possible to solve the data in a space group of accordingly higher symmetry.

PLAT112_ALERT_2_B ADDSYM Detects New (Pseudo) Symm. Elem c/2 100 %Fit

Author Response: This sample is a small distortion superstructure of a parent phase with c/2 phase. The closeis relationship to the higher symmetry relative phase is what is detected by ADDSYMM. The reflections due to the doubling of the c axis can be clearly seen in the raw data and are not seen in the datasets of the c/2 phase. We are, therefore, able to confidently assign the supercell. We note that there is no mechanism by which these intensities can arise from multiple scattering. Similarly, while the observed twinning would produce reflections with l odd (i.e. reflections indicative of cell doublinge) for h odd, we also observe reflections with l odd for h even. Thus, twinning of the primary phase cannot account for the observed diffraction. We note finally that, although the diffraction falls on a centred lattice of orthorhombic metric symmetry, it was not possible to solve the data in a space group of accordingly higher symmetry.

PLAT113_ALERT_2_B ADDSYM Suggests Possible Pseudo/New Space-group P21/m Check
Check Model Parameter Symmetry for Reflection Data Support

Author Response: This is the present space group, while the alert itself relates to the observation of a potential solution in a c/2 reduced cell, as discussed.

```
PLAT911_ALERT_3_B Missing FCF Refl Between Thmin & STh/L=    0.600        339 Report
                2  0  0,   3  0  0,   4  0  0,   0  2  0,   1  3  0,   0 10  0,
                1  0  1,   2  3  1,  -3  5  1,  -2  0  2,  -1  0  2,   0  0  2,
                0  1  2,   1  2  2,  -1  3  2,  -1  4  2,   1  6  2,   2  6  2,
               -3  0  3,   0  0  3,   1  0  3,   0  1  3,  -1  3  3,   0  3  3,
                2  4  3,  -1  5  3,   0  7  3,   0  0  4,   1  0  4,   0  1  4,
                   (  309 More NOT listed: see .ckf listing file)
```

Author Response: Only two crystallites viable for merging were obtained of this phase during the experiment, it being by far the minor phase and also exhibiting twinning. Between that, some preferred orientation, some outlier omission, and the relatively low symmetry, a data completeness of only 78.8% is obtained.

Alert level C

```
RINTA01_ALERT_3_C  The value of Rint is greater than 0.12
            Rint given   0.150
PLAT020_ALERT_3_C The Value of Rint is Greater Than 0.12 .........      0.150 Report
PLAT042_ALERT_1_C Calc. and Reported MoietyFormula Strings  Differ     Please Check
              Calc: C6 H4 Cl2 Cr N2 S
              Rep.: 2(C3 H2 Cl Cr0.5 N S0.5)
PLAT082_ALERT_2_C High R1 Value ..................................       0.14 Report
PLAT084_ALERT_3_C High wR2 Value (i.e. > 0.25) ...................       0.34 Report
PLAT250_ALERT_2_C Large U3/U1 Ratio for <U(i,j)> Tensor(Resd    1)        2.7 Note
PLAT250_ALERT_2_C Large U3/U1 Ratio for <U(i,j)> Tensor(Resd    2)        2.3 Note
PLAT351_ALERT_3_C Long   C-H (X0.96,N1.08A)  C12      - H12      .       1.16 Ang.
PLAT351_ALERT_3_C Long   C-H (X0.96,N1.08A)  C13      - H13      .       1.16 Ang.
PLAT351_ALERT_3_C Long   C-H (X0.96,N1.08A)  C22      - H22      .       1.16 Ang.
PLAT351_ALERT_3_C Long   C-H (X0.96,N1.08A)  C23      - H23      .       1.16 Ang.
PLAT767_ALERT_4_C INS Embedded LIST 6 Instruction Should be LIST 4     Please Check
PLAT906_ALERT_3_C Large K Value in the Analysis of Variance ......      9.187 Check
PLAT906_ALERT_3_C Large K Value in the Analysis of Variance ......      3.198 Check
PLAT906_ALERT_3_C Large K Value in the Analysis of Variance ......      2.237 Check
PLAT906_ALERT_3_C Large K Value in the Analysis of Variance ......      2.039 Check
PLAT913_ALERT_3_C Missing # of Very Strong Reflections in FCF ....         10 Note
                2  0  0,   0  0  2,  -1  3  2,   1  0  4,  -1  0  6,   1  0  6,
                1  2  6,   0  3  8,   0  4  8,   0  0 10,
```

Alert level G

```
ABSMU01_ALERT_1_G  Calculation of _exptl_absorpt_correction_mu
                not performed for this radiation type.
PLAT002_ALERT_2_G Number of Distance or Angle Restraints on AtSite         14 Note
PLAT003_ALERT_2_G Number of Uiso or U(i,j)  Restrained non-H-Atoms         14 Report
PLAT004_ALERT_5_G Polymeric Structure Found with Maximum Dimension          1 Info
PLAT019_ALERT_1_G _diffrn_measured_fraction_theta_full/*_max < 1.0      0.988 Report
PLAT072_ALERT_2_G SHELXL First  Parameter in WGHT  Unusually Large       0.13 Report
PLAT073_ALERT_1_G H-atoms ref., but hydrogen treatment Reported as     constr Check
```

```
PLAT116_ALERT_2_G ADDSYM Included (Pseudo) Lattice Translation ...     Please Check
PLAT176_ALERT_4_G The CIF-Embedded .res File Contains SADI Records         10 Report
PLAT178_ALERT_4_G The CIF-Embedded .res File Contains SIMU Records          2 Report
PLAT187_ALERT_4_G The CIF-Embedded .res File Contains RIGU Records          1 Report
PLAT190_ALERT_3_G A Non-default RIGU Restraint Value for First Par     0.0010 Report
PLAT190_ALERT_3_G A Non-default RIGU Restraint Value for SecondPar     0.0010 Report
PLAT199_ALERT_1_G Reported _cell_measurement_temperature ..... (K)        293 Check
PLAT200_ALERT_1_G Reported   _diffrn_ambient_temperature ..... (K)        293 Check
PLAT333_ALERT_2_G Large Aver C6-Ring C-C Dist C11      -C11_c    .       1.42 Ang.
PLAT335_ALERT_2_G Check Large C6 Ring C-C Range C11      -C11_c          0.15 Ang.
PLAT768_ALERT_4_G RES Embedded Explicitly Supplied Scattering Data          6 Note
PLAT769_ALERT_4_G CIF Embedded Explicitly Supplied Scattering Data          6 Note
PLAT794_ALERT_5_G Tentative Bond Valency for Cr1       (II)      .       1.96 Info
PLAT794_ALERT_5_G Tentative Bond Valency for Cr2       (II)      .       2.09 Info
PLAT802_ALERT_4_G CIF Input Record(s) with more than 80 Characters          4 Info
PLAT860_ALERT_3_G Number of Least-Squares Restraints ............         106 Note
PLAT870_ALERT_4_G ALERTS Related to Twinning Effects Suppressed ..          ! Info
PLAT910_ALERT_3_G Missing FCF Reflection(s) Below Theta(Min)[Deg]=       0.07 Note
                0  0  1,
PLAT912_ALERT_4_G Missing # of FCF Reflections Above STh/L=  0.600        108 Note
PLAT933_ALERT_2_G Number of HKL-OMIT Records in Embedded .res File         67 Note
               -5  2  8,  -3  0  3,  -3  5  1,  -3 12  2,  -3 12  6,  -2  0  2,
               -2  2  6,  -2  4  6,  -2  4 13,  -2  6 14,  -2  7  4,  -2  7 14,
               -1  0  2,  -1  3  2,  -1  3  3,  -1  3  9,  -1  4  2,  -1  4  9,
               -1  5  3,  -1  5 10,  -1  5 11,  -1  7 13,   0  1  2,   0  2  0,
                0  2  4,   0  3  3,   0  3  8,   0  4  8,   0  4 10,   0  6  4,
                0  6  6,   0  7  3,   0  7 14,   0  8  8,   0 10  0,   0 10 20,
                0 12  6,   1  0  1,   1  0  3,   1  2  2,   1  2  6,   1  3  0,
                1  3  7,   1  4  4,   1  4  6,   1  4  9,   1  5 11,   1  5 12,
                1  6  2,   1  6  6,
                   (   17 More NOT listed: see .ckf listing file)
PLAT941_ALERT_3_G Average HKL Measurement Multiplicity ..........         4.5 Low
PLAT948_ALERT_5_G Externally Supplied Scattering Factors       CIF          6 Note
PLAT948_ALERT_5_G Externally Supplied Scattering Factors       RES          6 Note
PLAT958_ALERT_1_G Calculated (ThMax) and Actual (FCF) Lmax Differ.          3 Units
PLAT969_ALERT_5_G The 'Henn et al.' R-Factor-gap value ..........      3.359 Note
              Predicted wR2: Based on SigI**2 10.00 or SHELX Weight 32.26
PLAT994_ALERT_1_G SHELXL .ins Contains no or MERG 0 Instruction ..          ! Note
```

---

   0 **ALERT level A** = Most likely a serious problem - resolve or explain
   4 **ALERT level B** = A potentially serious problem, consider carefully
  17 **ALERT level C** = Check. Ensure it is not caused by an omission or oversight
  33 **ALERT level G** = General information/check it is not something unexpected

   8 ALERT type 1 CIF construction/syntax error, inconsistent or missing data
  13 ALERT type 2 Indicator that the structure model may be wrong or deficient
  18 ALERT type 3 Indicator that the structure quality may be low
   9 ALERT type 4 Improvement, methodology, query or suggestion
   6 ALERT type 5 Informative message, check

It is advisable to attempt to resolve as many as possible of the alerts in all categories. Often the minor alerts point to easily fixed oversights, errors and omissions in your CIF or refinement strategy, so attention to these fine details can be worthwhile. It is up to the individual to critically assess their own results and, if necessary, seek expert advice.

PLATON version of 15/01/2026; check.def file version of 02/01/2026

---

duplicate check

No duplication found

Datablock m2_516_517_L072_R07 - ellipsoid plot

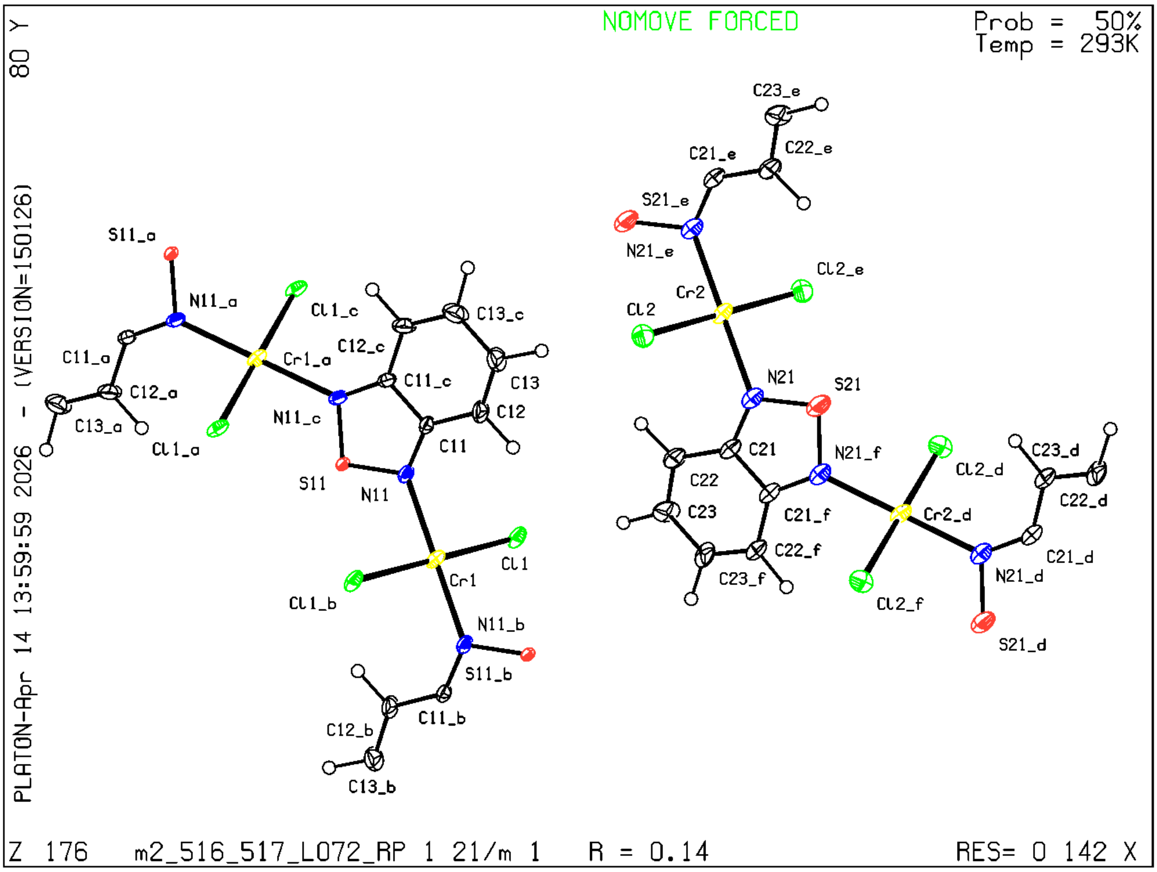

Supplement: Supplementary file 2 [file cm6c00516_si_002.zip › cifs/2023NCS0690_CRJPLE_ab2c_P21m__checkcif.pdf]
