# Supplementary material for: Enhancing Superexchange through Frontier Orbital Engineering in a van der Waals Metal–Organic Magnet
Source: Chem Mater. 2026 May 26;38(11):5649–57. doi: 10.1021/acs.chemmater.6c00516 (PMC13255171; doi:10.1021/acs.chemmater.6c00516)

checkCIF/PLATON report

Structure factors have been supplied for datablock(s) m4_513_4_5_5

THIS REPORT IS FOR GUIDANCE ONLY. IF USED AS PART OF A REVIEW PROCEDURE FOR PUBLICATION, IT SHOULD NOT REPLACE THE EXPERTISE OF AN EXPERIENCED CRYSTALLOGRAPHIC REFEREE.

No syntax errors found. CIF dictionary Interpreting this report

Datablock: m4_513_4_5_5

```
Bond precision:    C-C = 0.0087 A            Wavelength=0.02510

Cell:              a=3.7180(6)     b=12.9099(13)         c=8.7866(19)
                   alpha=90        beta=96.149(17)       gamma=90
Temperature:       293 K

                  Calculated                  Reported
Volume            419.32(12)                  419.32(12)
Space group       P 21/m                      P 1 21/m 1
Hall group        -P 2yb                      -P 2yb
Moiety formula    C6 H4 Cl2 Cr N2 S           C6 H4 Cl2 Cr N2 S
Sum formula       C6 H4 Cl2 Cr N2 S           C6 H4 Cl2 Cr N2 S
Mr                259.07                      259.08
Dx,g cm-3         2.052                       2.052
Z                 2                           2
Mu (mm-1)         0.000                       0.000
F000              0.0                         86.8
F000'             255.96
h,k,lmax          5,20,13                     5,20,13
Nref              1758                        1643
Tmin,Tmax
Tmin'

Correction method= Not given

Data completeness= 0.935           Theta(max)= 1.130

R(reflections)= 0.1629( 1236)                          wR2(reflections)=
                                                       0.3827( 1643)
S = 1.029                 Npar= 63
```

The following ALERTS were generated. Each ALERT has the format
test-name_ALERT_alert-type_alert-level.
Click on the hyperlinks for more details of the test.

Alert level A

RINTA01_ALERT_3_A The value of Rint is greater than 0.25
Rint given 0.260

Author Response: The data constitue the merging of 4 electron diffraction data- sets and the Rint of the resulting dataset is fairly good and typical. This is owed to differing impacts of dynamical effects across the various samples serving to significantly reduce internal agreement. These are expected to average out in merging to improve ultimate model statistics.

PLAT020_ALERT_3_A The Value of Rint is Greater Than 0.12 .... 0.260 Report

Author Response: The data constitue the merging of 4 electron diffraction data- sets and the Rint of the resulting dataset is fairly good and typical. This is owed to differing impacts of dynamical effects across the various samples serving to significantly reduce internal agreement. These are expected to average out in merging to improve ultimate model statistics.

Alert level B

PLAT029_ALERT_3_B _diffrn_measured_fraction_theta_full value Low . 0.957 Why?

Author Response: Despite merging 4 datasets to form these data, a modest preferred orientation remained that made obtaining full completeness for this (P21/m) model, while a further 44 reflections were omitted as outliers / erroneous. This small loss of completeness is not anticipated to have any meaningful impact on the analysis in the context of the present study.

PLAT082_ALERT_2_B High R1 Value .... 0.16 Report

Author Response: The data constitue the merging of 4 electron diffraction data-sets and are treated in the kinematic approximation using an extinction correction to account for the broad effects of multiple scattering. The R1 of the resulting dataset is fairly good and typical in this context. Care has been taken to reject reflections that are more significantly affected by these effects and ensure that the model is robust in this context.

```
PLAT084_ALERT_3_B High wR2 Value (i.e. > 0.25) ..................       0.38 Report
```

Author Response: The data constitue the merging of 4 electron diffraction data-sets and are treated in the kinematic approximation using an extinction correction to account for the broad effects of multiple scattering. The wR2 of the resulting dataset is fairly good and typical in this context, slightly higher than expected from the R1 owing to the more varied and proportionally significant impact of multiple scattering on weak data. Care has been taken to reject reflections that are more significantly affected by these effects and ensure that the model is robust in this context.

Alert level C

```
PLAT250_ALERT_2_C Large U3/U1 Ratio for <U(i,j)> Tensor(Resd    1)        3.1 Note
PLAT341_ALERT_3_C Low Bond Precision on  C-C Bonds ...............    0.00875 Ang.
PLAT351_ALERT_3_C Long   C-H (X0.96,N1.08A)  C2       - H2       .       1.15 Ang.
PLAT351_ALERT_3_C Long   C-H (X0.96,N1.08A)  C3       - H3       .       1.14 Ang.
PLAT431_ALERT_2_C Short Inter HL..A Contact  Cl1      ..S1       .       3.37 Ang.
                                                -1+x,y,z  =      1_455 Check
PLAT906_ALERT_3_C Large K Value in the Analysis of Variance ......     53.425 Check
PLAT906_ALERT_3_C Large K Value in the Analysis of Variance ......      7.965 Check
PLAT906_ALERT_3_C Large K Value in the Analysis of Variance ......      4.187 Check
PLAT906_ALERT_3_C Large K Value in the Analysis of Variance ......      2.348 Check
PLAT911_ALERT_3_C Missing FCF Refl Between Thmin & STh/L=    0.600         33 Report
                 2  0  0,   3  0  0,   0  2  0,   0  4  0,   2  4  0,  -2  0  1,
                -1  0  1,   1  0  1,  -2  1  2,   2  2  2,   0  0  3,   1  0  3,
                 1  0  4,   1  2  4,   0  4  4,  -1  7  4,  -4  0  5,  -1  0  5,
                 0  0  5,   1  0  5,   0  5  5,   1  7  5,  -3  0  6,   0  0  6,
                 1  0  6,   2  0  6,   0  0  7,   2  0  7,   0  0  8,   2  0  8,
                 0  0  9,   1  0  9,  -1  4 10,
```

Alert level G

```
ABSMU01_ALERT_1_G  Calculation of _exptl_absorpt_correction_mu
                 not performed for this radiation type.
PLAT002_ALERT_2_G Number of Distance or Angle Restraints on AtSite          4 Note
PLAT004_ALERT_5_G Polymeric Structure Found with Maximum Dimension          1 Info
PLAT072_ALERT_2_G SHELXL First  Parameter in WGHT  Unusually Large       0.12 Report
PLAT073_ALERT_1_G H-atoms ref., but hydrogen treatment Reported as     constr Check
PLAT176_ALERT_4_G The CIF-Embedded .res File Contains SADI Records          1 Report
PLAT199_ALERT_1_G Reported _cell_measurement_temperature ..... (K)        293 Check
PLAT200_ALERT_1_G Reported   _diffrn_ambient_temperature ..... (K)        293 Check
PLAT768_ALERT_4_G RES Embedded Explicitly Supplied Scattering Data     Please Note
PLAT769_ALERT_4_G CIF Embedded Explicitly Supplied Scattering Data     Please Note
PLAT794_ALERT_5_G Tentative Bond Valency for Cr1       (II)       .       2.03 Info
PLAT802_ALERT_4_G CIF Input Record(s) with more than 80 Characters          1 Info
PLAT860_ALERT_3_G Number of Least-Squares Restraints ............           2 Note
PLAT910_ALERT_3_G Missing # of FCF Reflection(s) Below Theta(Min).          1 Note
                 0  0  1,
PLAT912_ALERT_4_G Missing # of FCF Reflections Above STh/L=  0.600         64 Note
PLAT913_ALERT_3_G Missing # of Very Strong Reflections in FCF ....          3 Note
                 2  0  0,   0  0  1,   1  0  3,
PLAT933_ALERT_2_G Number of HKL-OMIT Records in Embedded .res File         44 Note
```

```
              -5  0  3,  -5  1  3,  -5  6  6,  -5  8  3,  -5  9  3,  -4  0  5,
              -4  4  9,  -4  5  9,  -4  6  9,  -3  0  6,  -3  0 11,  -2  0  1,
              -2  1  2,  -1  0  1,  -1  0  5,  -1  4 10,  -1  7  4,   0  0  1,
               0  0  3,   0  0  5,   0  0  6,   0  0  7,   0  0  8,   0  0  9,
               0  2  0,   0  4  0,   0  4  4,   0  5  5,   1  0  1,   1  0  9,
               1  2  4,   1  7  5,   2  0  0,   2  1  9,   2  1 10,   2  2  2,
               2  4  0,   3  0  0,   3  5 10,   4  1  5,   4  1  7,   4  3  7,
               4 14  2,   5  3  0,
PLAT969_ALERT_5_G The 'Henn et al.' R-Factor-gap value ..........      2.864 Note
              Predicted wR2: Based on SigI**2 13.36 or SHELX Weight 37.19
PLAT978_ALERT_2_G Number C-C Bonds with Positive Residual Density.         0 Info
```

```
  2 ALERT level A = Most likely a serious problem - resolve or explain
  3 ALERT level B = A potentially serious problem, consider carefully
 10 ALERT level C = Check. Ensure it is not caused by an omission or oversight
 19 ALERT level G = General information/check it is not something unexpected

  4 ALERT type 1 CIF construction/syntax error, inconsistent or missing data
  7 ALERT type 2 Indicator that the structure model may be wrong or deficient
 15 ALERT type 3 Indicator that the structure quality may be low
  5 ALERT type 4 Improvement, methodology, query or suggestion
  3 ALERT type 5 Informative message, check
```

It is advisable to attempt to resolve as many as possible of the alerts in all categories. Often the minor alerts point to easily fixed oversights, errors and omissions in your CIF or refinement strategy, so attention to these fine details can be worthwhile. In order to resolve some of the more serious problems it may be necessary to carry out additional measurements or structure refinements. However, the purpose of your study may justify the reported deviations and the more serious of these should normally be commented upon in the discussion or experimental section of a paper or in the "special_details" fields of the CIF. checkCIF was carefully designed to identify outliers and unusual parameters, but every test has its limitations and alerts that are not important in a particular case may appear. Conversely, the absence of alerts does not guarantee there are no aspects of the results needing attention. It is up to the individual to critically assess their own results and, if necessary, seek expert advice.

Publication of your CIF in IUCr journals

A basic structural check has been run on your CIF. These basic checks will be run on all CIFs submitted for publication in IUCr journals (*Acta Crystallographica*, *Journal of Applied Crystallography*, *Journal of Synchrotron Radiation*); however, if you intend to submit to *Acta Crystallographica Section C* or *E* or *IUCrData*, you should make sure that full publication checks are run on the final version of your CIF prior to submission.

Publication of your CIF in other journals

Please refer to the *Notes for Authors* of the relevant journal for any special instructions relating to CIF submission.

PLATON version of 02/02/2025; check.def file version of 02/02/2025

Datablock m4_513_4_5_5 - ellipsoid plot

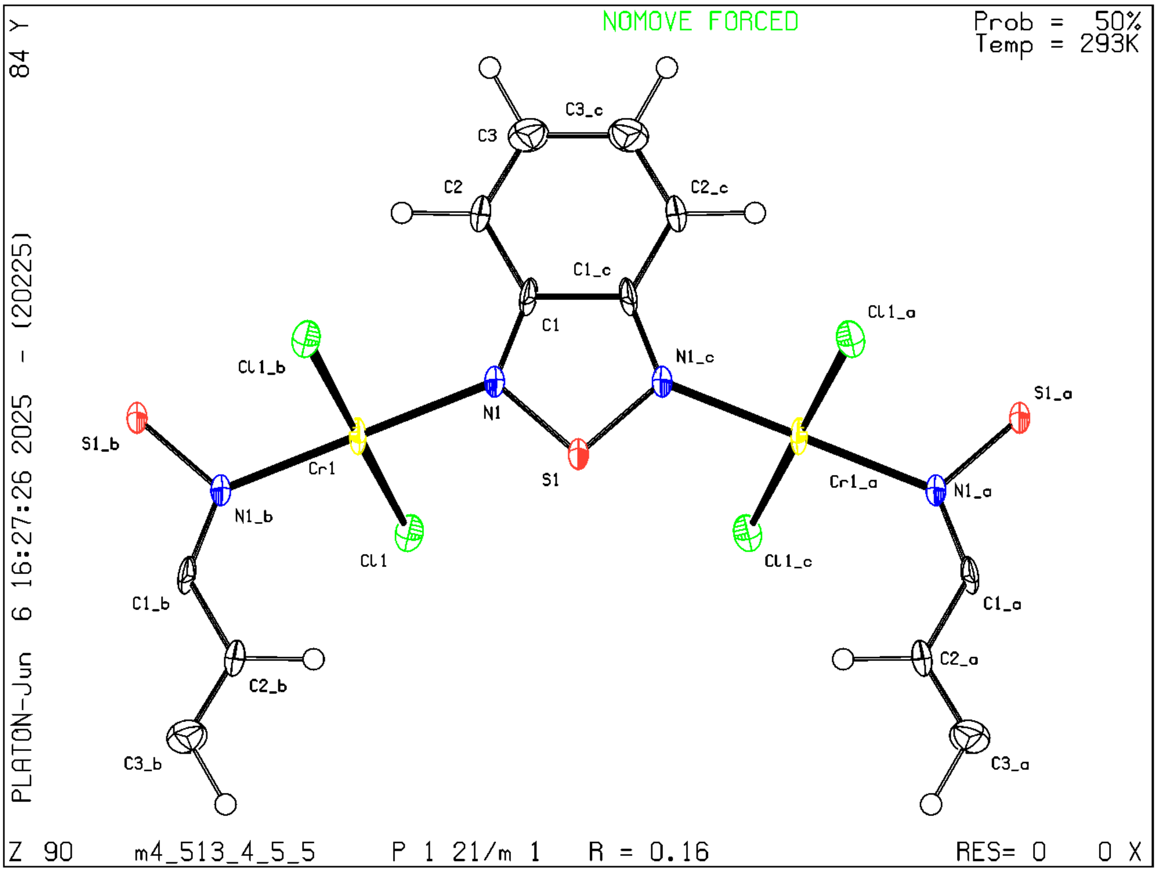

Supplement: Supplementary file 2 [file 2023NCS0690_CRJPLE_abc_P21m__checkcif.pdf › cifs/2023NCS0690_CRJPLE_abc_P21m__checkcif.pdf]
